# Antibody–Drug Conjugates Targeting HER2 and Trop-2: A New Force in Precision Treatment for Solid Tumors

**DOI:** 10.3390/ph19081194

**Published:** 2026-07-29

**Authors:** Zhaoling Jiang, Chen Mei, Xueze Lyu, Zhenyi Liu, Zhihua Li, Baozhu Xing, Ying Liu, Gebin Li, Hongjun Wang

**Affiliations:** 1 College of Animal Science and Technology, Beijing University of Agriculture, Beijing 102206, China; 2 Institute of Animal Husbandry and Veterinary Medicine, Beijing Academy of Agriculture and Forestry Sciences, Beijing 100097, China; meichen@baafs.net.cn (C.M.);; 3 Beijing General Animal Husbandry Station, Beijing 100107, China; 4 Small Animal Department, College of Veterinary Medicine, China Agricultural University, Beijing 100193, China

**Keywords:** antibody–drug conjugates, HER2, Trop-2, solid tumors

## Abstract

Human epidermal growth factor receptor 2 (HER2) and trophoblast cell surface antigen 2 (Trop-2) are tumor-associated antigens widely overexpressed in multiple malignant tumors, which drive malignant proliferation, invasion, metastasis, and therapeutic resistance by activating key downstream signaling pathways. Antibody–drug conjugates (ADCs) targeting HER2 and Trop-2 leverage their antigen-specific binding capacity to achieve precise targeted delivery of cytotoxic drugs, representing a significant breakthrough in solid tumor therapy. This review systematically outlines the biological functions and carcinogenic mechanisms of HER2 and Trop-2, focusing on the latest research and development progress of related ADCs. We also summarize and analyze key clinical data and application prospects in breast cancer (BC), gastric cancer (GC), non-small cell lung cancer (NSCLC), and urothelial carcinoma (UC), while also delving into the challenges and future directions within this field.

## 1. Introduction: The Rise of Antibody–Drug Conjugates in Precision Oncology

Antibody–drug conjugates (ADCs) represent a class of targeted therapeutics engineered by linking monoclonal antibodies to potent cytotoxic drugs, enabling selective destruction of tumor cells. They have emerged as a significant research focus within contemporary oncology [1]. ADCs demonstrate significant therapeutic potential across multiple malignant solid tumors, with particularly widespread application in breast cancer (BC), gastric cancer (GC), non-small cell lung cancer (NSCLC), and urothelial carcinoma (UC). Among these, human epidermal growth factor receptor 2 (HER2) and trophoblast cell surface antigen 2 (Trop-2) serve as key target antigens that are overexpressed in numerous epithelial tumors and are closely associated with tumor proliferation, invasion, and metastasis. Presently, the U.S. Food and Drug Administration (FDA) has approved multiple ADC drugs for market release, whilst a substantial number of candidate drugs are undergoing clinical trials at various stages, indicating broad application prospects.

Compared with using payload chemotherapy alone, ADCs deliver highly potent cytotoxic payloads precisely to tumor cells via targeted antibodies, significantly reducing toxicities to normal tissues. This targeting mechanism broadens the therapeutic window and enhances antitumor efficacy. Moreover, ADCs remain stable in circulation and only release the active drug inside target cells, further reducing systemic exposure.

In recent years, agents such as trastuzumab deruxtecan (T-DXd), targeting HER2, and sacituzumab govitecan (SG), targeting Trop-2, have demonstrated favorable efficacy in clinical trials. T-DXd not only demonstrates significant efficacy in HER2-positive BC, but also exhibits profound and durable antitumor activity in patients with HER2-low metastatic BC, remaining effective even after multiple prior treatments [2]. Subsequent studies have confirmed the efficacy of SG in the treatment of triple-negative breast cancer (TNBC) and HR-positive/HER2-negative breast cancer; therefore, SG can be used to treat these conditions [3].

Despite demonstrating remarkable therapeutic value, the clinical application of ADCs faces numerous challenges. Foremost among these is the issue of resistance, which may arise from antigen loss, impaired intracellular drug transport, and enhanced extracellular drug efflux [4]. Secondly, the use of ADCs is associated with specific toxicities, potentially linked to the antibody–drug conjugation methodology, drug release mechanisms, and the tumor microenvironment [5]. Consequently, current research priorities include developing more specific and safer ADCs, optimizing the design of linkers and cytotoxins, and exploring biomarker-guided personalized treatment strategies.

**Figure 1:**
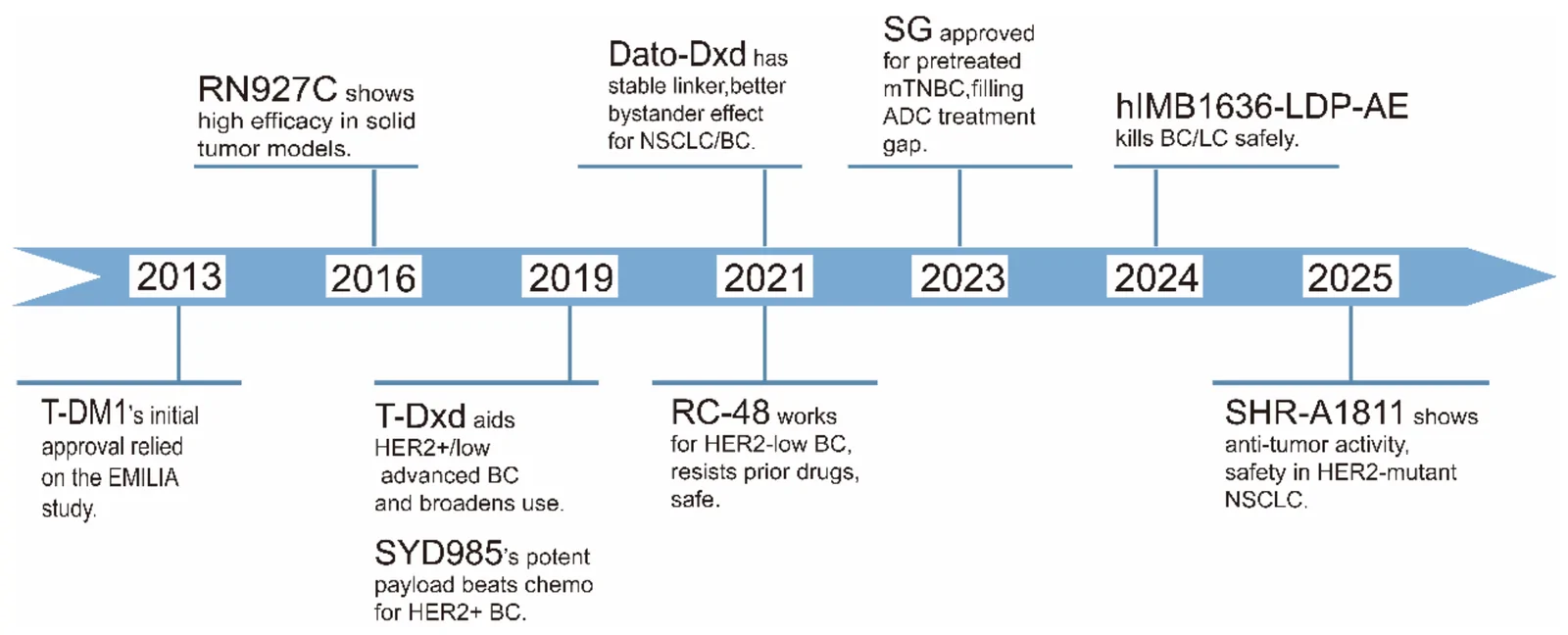
This timeline illustrates the development progress of antibody–drug conjugates targeting HER2 and Trop-2.

As clinical evidence accumulates, ADCs targeting HER2 and Trop-2 are increasingly integrated into standard treatment regimens for a variety of malignancies. Nevertheless, several core challenges limit current clinical applications of ADCs. First, patient selection remains a pressing issue: how can we move beyond conventional immunohistochemistry (IHC) and fluorescence in situ hybridization (FISH) testing to accurately identify patients who will derive benefit? Moreover, there is no consensus on expression thresholds for HER2-low and Trop-2. Second, toxicity management requires refinement: the distinctive toxicity profile of ADCs, including interstitial pneumonitis, ocular toxicity, and neuropathy, has important implications for clinical decision-making and health-related quality of life. In addition, payload cross-resistance is an increasingly prominent challenge, particularly with the widespread use of TOP1 inhibitor-based ADCs. Future research should not only elucidate resistance mechanisms of ADCs in greater depth but also actively explore combinations with other therapeutic modalities, such as immune checkpoint inhibitors, other targeted therapies, and conventional chemotherapy, to develop more effective and precise treatment strategies. This review aims to provide a theoretical foundation and practical directions for optimizing clinical treatment protocols and advancing personalized medicine by systematically summarizing the mechanisms of action, resistance pathways, and clinical progress of ADCs across various tumor types (Figure 1).

**Figure 2:**
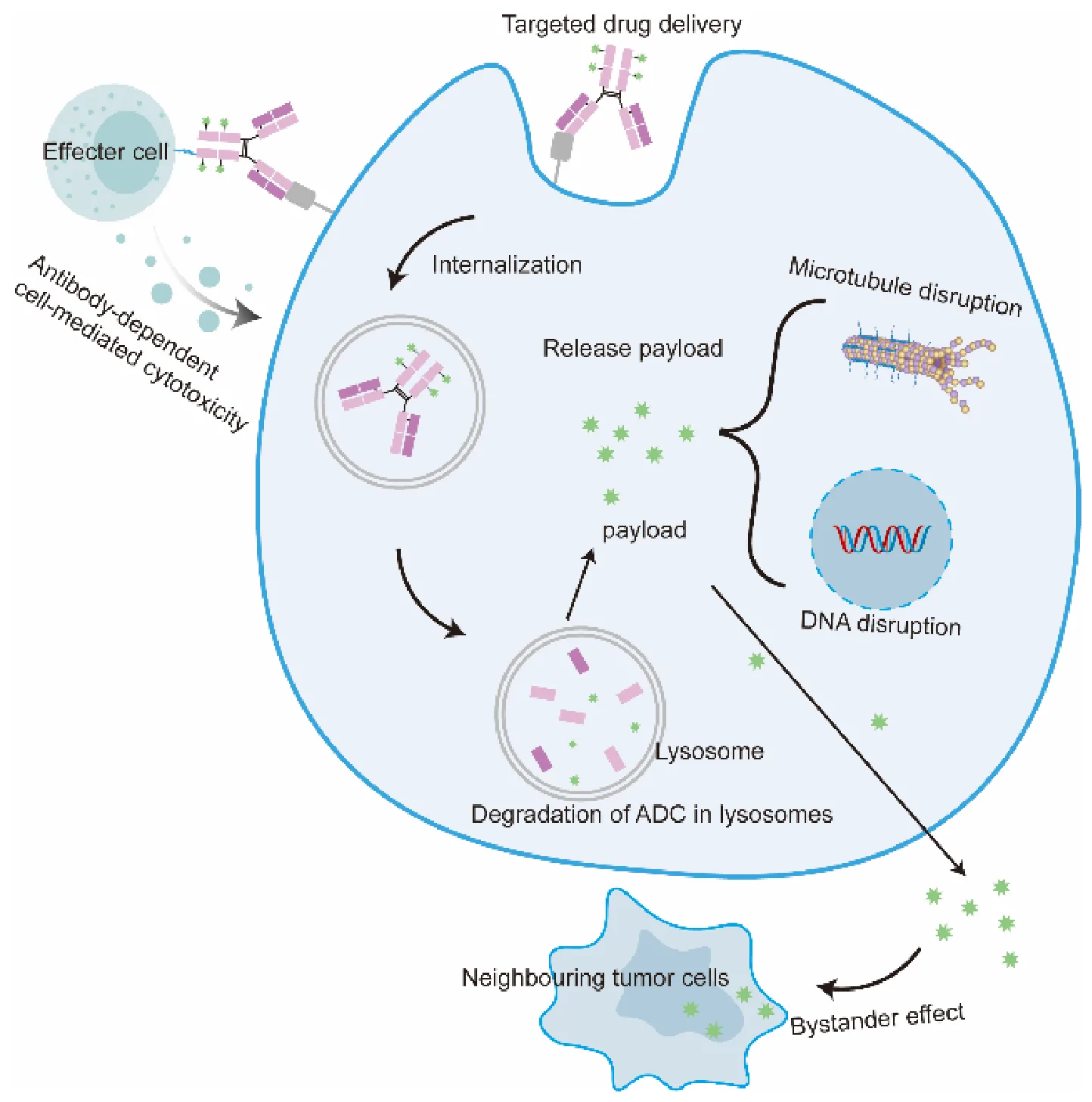
The endocytic–lysosomal pathway and bystander effects of antibody–drug conjugates in inducing tumor cell death.

The concept of ADCs, first proposed by Paul Ehrlich in 1913 [5], is based on a tripartite construct comprising a monoclonal antibody, a linker, and a cytotoxic payload. The antibody must balance high target affinity with low immunogenicity, a long circulating half-life, and systemic stability to ensure efficient tumor accumulation and internalization [6]. The Fab fragment mediates antigen binding and may modulate receptor signaling. The Fc region engages Fc-mediated effector functions, including antibody-dependent cellular cytotoxicity, antibody-dependent cellular phagocytosis, and complement-dependent cytotoxicity, thereby augmenting antitumor activity [6]. ADCs have been predominantly developed on chimeric or humanized IgG1 or IgG4 backbones [7,8,9]. The linker must remain stable in circulation to prevent off-target toxicity yet be efficiently cleaved intracellularly to release the payload. Internalization typically occurs via receptor-mediated endocytosis, forming early endosomes that mature into late endosomes and fuse with lysosomes. In the acidic lysosomal milieu (pH~4.5–5.0) with abundant proteolytic activity, the antibody portion is degraded, and the cleavable linker is cleaved, releasing the cytotoxic payload (Figure 2). Cleavable linkers release membrane-permeable payloads, generating a bystander effect that enhances efficacy against antigen-heterogeneous tumors [10]. These hydrophobic payloads (DXd and SN-38) can diffuse across cell membranes to affect adjacent tumor cells irrespective of their target antigen expression, helping eradicate antigen-heterogeneous tumor cells and improving therapeutic outcomes. However, cleavable linkers carry the risk of off-target release, contributing to toxicities such as interstitial pneumonitis (T-DXd) or hematologic toxicity and diarrhea SG [11,12]. The payloads used in ADCs are diverse, encompassing alkaloids, alkylating agents, antimetabolites, antitumor antibiotics, topoisomerase inhibitors, and mitotic inhibitors. Currently approved ADC payloads predominantly include microtubule inhibitors (e.g., monomethyl auristatin E (MMAE) and DM1), topoisomerase I inhibitors (e.g., DXd and SN-38), and DNA-damaging agents (e.g., carmustine and pyrrolobenzodiazepine (PBD) dimers), among others [13]. These features position ADCs as a unique drug-delivery platform [14]. ADC design has evolved across four generations toward greater homogeneity, stability, and efficacy [15]. The first ADC to receive FDA approval was gemtuzumab ozogamicin, developed by Wyeth and introduced in 2000 for treating acute myeloid leukemia. This compound consists of an anti-CD33 monoclonal antibody conjugated to ozogamicin, the deployed cytotoxic payload. Subsequent studies, however, indicated that its combination with conventional chemotherapy failed to significantly improve overall survival while being associated with high-grade toxicity. Consequently, Pfizer, after acquiring Wyeth, voluntarily withdrew the drug from the market in 2010 [14]. These milestones underscore both the potential and the challenges of ADCs and set the stage for the subsequent progress in ADC design and clinical development.

## 2. The Evolution of ADCs

The evolution of ADC design has centered on optimizing each of its three core components—antibody, linker, and payload—to improve therapeutic index. Linkers are classified as cleavable or non-cleavable. Non-cleavable linkers rely on the lysosomal degradation of the antibody for payload release, generally resulting in different safety and efficacy profiles.

Noncleavable linkers exhibit greater stability in plasma, facilitating a broader therapeutic window and reduced off-target toxicity risk, as their payloads are released only after endocytosis and intracellular enzymatic cleavage [16]. Different payloads exhibit distinct toxicological profiles: for example, the microtubule inhibitors MMAE and DM1 often cause neuropathy, whereas the topoisomerase I inhibitors DXd and SN-38 are associated with interstitial pneumonia, hematological toxicity, and diarrhea. These differences represent key clinical considerations that must be addressed during ADC design [17].

Payload design should consider not only cytotoxic potency but also stability, solubility, and binding affinity to the linker [18]. Subsequently, second-generation ADCs emerged, including brentuximab vedotin (targeting CD30), approved in 2011, and trastuzumab emtansine (T-DM1), approved in 2013 (Figure 1). These agents employ humanized or fully human antibodies, significantly reducing immunogenicity and improving pharmacokinetic properties, representing major advances in ADC technology [19]. Third-generation ADCs further employed hydrophilic linkers to counteract the adverse effects of certain highly hydrophobic cytotoxic drugs such as PBD. This effectively improved the solubility of ADC molecules, reduced aggregation tendencies, and thereby prolonged their circulation time and enhanced tumor tissue penetration [20]. Building upon this foundation, fourth-generation ADCs further optimized the drug–antibody ratio (DAR) to achieve more uniform and controllable drug distribution [21]. High-DAR designs aim to increase the payload per antibody to enhance potency, but they also place higher demands on linker stability to prevent ADC aggregation and toxicity from premature payload release. In contrast, low-DAR designs offer a wider safety margin but may have lower potency. Achieving a balance between the efficacy of high-DAR designs and the safety of low-DAR designs is a central challenge in fourth-generation ADC design, aiming to increase the concentration of payload released within tumor cells and thereby enhance antitumor efficacy. However, this involves a complex trade-off between reducing systemic exposure and off-target toxicity, since a higher DAR may be associated with a greater risk of toxicity. This represents a significant step forward for ADC technology in expanding the therapeutic window and broadening clinical applicability; however, whether these goals can ultimately be achieved remains to be demonstrated through precise molecular design and rigorous clinical validation.

Consequently, future research will prioritize ADC design, including the selection and linkage of antibodies, linkers, and payloads, to substantially enhance efficacy and safety, thereby addressing current clinical challenges. As clinical trials progress, ADCs are poised to play an increasingly pivotal role in future therapeutic frameworks, delivering more effective and precise treatments for patients.

## 3. The Biological Characteristics of HER2 and Its Role in Cancer

### 3.1. Biological Characteristics of HER2

HER2 is a human tyrosine kinase receptor encoded by the ERBB2 gene, lacking a ligand and also termed the erbB-2 oncogene. This protein is homologous to the neu protein in rodents; sequencing studies have confirmed extensive homology between the two, with the HER2 gene localized on chromosome 17 [22]. HER1, HER2, HER3, and HER4 collectively constitute the HER family. These receptors comprise three domains: intracellular, transmembrane, and extracellular. The extracellular region of HER2 lacks a known ligand and can be activated through homodimerization or heterodimerization [23]. Constitutive HER2 signaling primarily activates the phosphatidylinositol 3-kinase/protein kinase B/mammalian target of rapamycin (PI3K/AKT/mTOR) pathway, blocking apoptosis and promoting tumor progression [24]. Simultaneously, the mitogen-activated protein kinase/extracellular signal-regulated kinase (MAPK/ERK) pathway drives cell proliferation [25].

Although HER2 overexpression is a recognized oncogenic driver, HER2 protein expression does not always correlate with ERBB2 gene amplification [26]. This inconsistency is a key manifestation of HER2 heterogeneity, which includes spatial and temporal heterogeneity (for example, dynamic changes in HER2 status after treatment [27]). It is this variability that drives inconsistent responses to HER2-targeted therapies and can contribute to resistance [28]. HER2-low is defined as IHC 1+ or IHC 2+/FISH-negative, while HER2-ultralow refers to ≤10% of tumor cells exhibiting faint, incomplete membrane staining. Clinically, these patients show a significant response to T-DXd [29] and exhibit distinct immunomicroenvironmental characteristics and prognostic differences [30], offering a new direction for precision therapy in BC. Despite these advances, HER2 testing still faces challenges. Standard immunohistochemical methods are subjective and exhibit high inter-observer variability [31]. Furthermore, HER2 status is not static and may change as disease progresses or in response to therapeutic interventions. These dynamic changes further complicate the interpretation of test results and affect the accuracy of treatment decisions.

In summary, HER2 plays a complex and crucial role in tumorigenesis, and its structural and signaling characteristics render it a key target for targeted therapies. However, the advent of the ADCs era necessitates a fundamental reassessment of the definition of targetable HER2 status. Recognition of HER2 heterogeneity, the redefinition of low- and ultralow-expression categories in clinical trials, and the acknowledged limitations of current IHC/FISH detection methods indicate that determining HER2 status is no longer a simple binary (positive or negative) classification. A deeper understanding of the dynamic expression and heterogeneity of HER2, coupled with the development of more sensitive and reproducible detection methods, will play a crucial role in delivering more effective personalized treatment regimens.

### 3.2. Applications of HER2-Targeted ADCs in Specific Cancers

HER2 plays a pivotal role in cancer treatment. Targeted therapies and ADCs developed to target this pathway have been validated through extensive clinical research and practice, demonstrating the ability to significantly prolong patient survival and enhance quality of life, thereby enabling personalized precision medicine. Currently, HER2-targeted ADCs primarily include T-DM1, T-DXd, disitamab vedotin (RC-48), trastuzumab duocarmazine (SYD985), MRG002, ARX788, and A166 [32].

#### 3.2.1. T-DM1

Within the precision diagnosis and treatment framework for BC, the accurate assessment of HER2 protein expression remains the cornerstone for formulating personalized therapeutic strategies. In the diagnosis and treatment of BC, the HER2 protein expression status is a critical factor. The current approach employs a combined assessment method utilizing IHC and FISH. IHC grading systems categorize HER2 protein expression into four levels: 3+, 2+, 1+, and 0 [33]. Accurate HER2 status determination plays a pivotal role in tailoring personalized treatment strategies for BC. Approximately 20% of BC patients exhibit HER2 gene amplification and protein overexpression, which activate multiple signaling pathways and promote tumor progression. HER2-positive BC is characterized by high invasiveness, rapid progression, a propensity for metastasis and recurrence, and short survival times. This discovery directly drove the clinical translation of trastuzumab, the first HER2-targeted monoclonal antibody [34].

Trastuzumab is the first humanized monoclonal antibody developed against HER2. It is a recombinant humanized monoclonal immunoglobulin G1 kappa (IgG1κ) antibody that specifically binds to extracellular domain IV of HER2 via its antigen-binding fragment (Fab), thereby blocking homodimerization/heterodimerization formation. This exerts a dual-pathway antitumor mechanism: it inhibits MAPK/ERK and PI3K/AKT signaling pathways to suppress cell proliferation [35], whilst simultaneously activating antibody-dependent cellular cytotoxicity via FcγR to recruit immune cells for tumor destruction [36].

Developed on this basis, T-DM1 became the first anti-HER2 ADC. It comprises trastuzumab chemically linked via an uncleavable thioether linker to the cytotoxic drug maytansinoid derivative (DM1) [37]. DM1 is a maytansinoid derivative with a DAR of 3.5, which disrupts mitosis by inhibiting tubulin polymerization, leading to tumor cell apoptosis [38]. Following antibody-mediated targeting to the HER2 receptor on tumor cell surfaces, T-DM1 enters cells via receptor-mediated endocytosis. It is subsequently transported to lysosomes for degradation, releasing the loaded DM1 metabolite (e.g., lysine-MCC-DM1). Subsequently, the solute carrier family 46 member three transporter (SLC46A3) on the lysosomal membrane transports the metabolite-containing trastuzumab into the cytoplasm [39]. This disrupts the microtubule network, impairs intracellular transport, and causes mitotic arrest, ultimately triggering cell cycle catastrophe and apoptosis (Table 1) [40]. In clinical practice, the indication for T-DM1 is defined as standard second-line treatment for patients with HER2-positive metastatic BC. This indication was definitively established by the landmark EMILIA trial (NCT00829166), which compared T-DM1 with capecitabine plus lapatinib. The results demonstrated a significant improvement in median progression-free survival (mPFS) (9.6 vs. 6.4 months; hazard ratio (HR) = 0.65) and median overall survival (mOS) (30.9 vs. 25.1 months; HR = 0.68) [41]. That remarkable efficacy can be attributed to the design of T-DM1. The non-cleavable linker ensures stability in circulation. The trastuzumab backbone provides high-affinity binding and internalization. The DM1 payload exerts potent cytotoxicity in cells with uniformly high HER2 expression. Collectively, these features confer a favorable therapeutic index in a population with homogeneous, high HER2 expression. A separate Phase III trial by the same sponsor further validated its efficacy in a multi-line treatment-experienced population: the T-DM1 group demonstrated a significant improvement in mPFS compared to physician’s choice regimens: 6.2 vs. 3.3 months (HR = 0.53, 95% CI 0.42–0.66) and mOS: 22.7 vs. 15.8 months [42]. These findings further cemented the pivotal role of T-DM1 in the treatment of HER2-positive metastatic BC.

It is noteworthy that the application of HER2-targeted therapy continues to expand. Beyond BC, HER2 abnormalities have been identified in various other cancers, including GC and NSCLC. Within the field of GC, approximately 20% of metastatic cases exhibit HER2 abnormalities, predominantly observed in intestinal-type and gastroesophageal junction cancers [43]. The GATSBY trial investigated the efficacy of T-DM1 in treating HER2-positive advanced GC in previously treated patients. Results demonstrated a significant correlation between efficacy and HER2 expression levels: in the IHC 3+ subgroup, T-DM1 showed comparable efficacy to taxanes (mOS: 9.5 vs. 8.3 months) [44]; however, efficacy was inferior in the IHC 2+/FISH+ subgroup (mOS: 5.2 vs. 9.2 months; HR = 1.53). This differential efficacy directly reflects the mechanistic limitations of T-DM1: its non-cleavable linker and impermeable DM1 payload cannot generate a bystander effect. Consequently, in the HER2-low and highly heterogeneous IHC 2+/FISH+ subgroup, a substantial fraction of tumor cells fail to internalize enough ADC, leading to sublethal intratumoral drug concentrations and poor prognosis. This pattern is consistent with observations in BC, further underscoring that T-DM1’s efficacy is confined to tumors with high, uniform HER2 expression [45]. In the NSCLC setting, T-DM1 activity varies according to the type of HER2 alteration. For patients with HER2 protein overexpression, efficacy is limited and observed only in the IHC 3+ cohort, with an objective response rate (ORR) of 20% [46]. Conversely, in approximately 3% of NSCLC patients harboring HER2 kinase domain mutations (predominantly exon 20 insertion mutations), T-DM1 demonstrated encouraging efficacy, with a phase II study reporting an ORR of 38.1% [47]. This limited activity is consistent with findings in gastric cancer. In gastric cancer, non-cleavable linkers and the absence of a bystander effect confine efficacy to HER2-high, homogeneous tumors. In NSCLC, due to high heterogeneity and medium-to-strong HER2 overexpression, few tumors meet this condition. By contrast, HER2 mutations may enhance sensitivity to targeted delivery by escaping Cbl-mediated ubiquitin degradation, thereby prolonging intratumoral retention of the ADC and increasing intracellular DM1 accumulation, while continuously activating the PI3K/AKT pathway to augment downstream signaling dependence.

T-DM1, as the pioneering HER2-targeted ADC, employs a thioether linker to achieve precise coupling between trastuzumab and the cytotoxic payload DM1 [45]. Its dual mechanism of action simultaneously blocks the HER2 signaling pathway and induces mitotic catastrophe in tumor cells [48]. Clinical studies confirm that T-DM1 treatment for BC, GC, and NSCLC significantly prolongs survival. This evidence establishes its position as standard second-line therapy [49]. Following this, T-DM1 uses a non-cleavable linker, which prevents a bystander effect. This directly limits its cytotoxic activity against tumors with high HER2 expression heterogeneity, including some HER2-low breast cancers and IHC 2+/FISH+ gastric cancers. Its relatively low DAR of 3.5 further constrains the delivered payload per administration. The payload DM1, a microtubule inhibitor, carries specific resistance risks, such as upregulation of the ABCB1 (MDR1) efflux transporter, which can lead to treatment failure.

In contrast, next-generation ADCs such as T-DXd employ a cleavable linker that releases a membrane-permeable payload, enabling bystander killing of antigen-negative tumor cells and thereby overcoming tumor heterogeneity. The DAR is higher, approximately 8, enabling greater payload delivery. Moreover, the payload DXd is a topoisomerase I inhibitor and is not subject to ABCB1-mediated resistance. It also provides a new, effective option for sequential therapy after resistance to T-DM1, thereby substantially improving efficacy.

The next section will detail how the structural design of T-DXd translates into its superior clinical efficacy.

#### 3.2.2. T-DXd

T-DXd is a next-generation HER2-targeted ADC that has been approved by the FDA for patients with advanced HER2-positive breast cancer who have previously received HER2-targeted therapies. Like T-DM1, it uses trastuzumab as its antibody backbone, but it exhibits significant differences in structure and mechanism of action. The DAR is approximately 8, and its payload is DXd, a topoisomerase I inhibitor. It employs a cleavable tetrapeptide linker that releases DXd through cleavage by cathepsins—lysosomal enzymes upregulated in many cancer cells. After HER2 binding, T-DXd undergoes receptor-mediated endocytosis into lysosomes, where the linker is cleaved, and DXd is released, interfering with DNA replication and repair to induce tumor cell death. These structural features underpin its enhanced clinical efficacy, as detailed below.

In BC, T-DXd has demonstrated remarkable efficacy both as a later-line therapy and, more importantly, as a new standard second-line option. In the later-line setting, DESTINY-Breast01 showed a confirmed ORR of 60.9% in patients who had previously received T-DM1 [50]. Subsequently, DESTINY-Breast03 directly compared T-DXd with T-DM1 in the second-line setting, reporting a mPFS of 28.8 months for T-DXd versus 6.8 months for T-DM1 (HR = 0.33)—a result that shifted T-DXd from a later-line option to the new standard second-line therapy. This unprecedented reduction in risk can be attributed, mechanism-wise, to the overall design advantages of T-DXd over T-DM1. The cleavable GGFG linker enables efficient DXd release within target cells, and a high DAR ensures high intratumoral payload concentrations. The strong bystander effect of DXd can overcome HER2 heterogeneity, addressing a key deficiency of T-DM1, which is limited by its non-cleavable linker and impermeable DM1 payload. Collectively, these features establish T-DXd as the new standard of care in the second-line setting. In a phase III trial by Hurvitz et al., the T-DXd group had a median follow-up of 28.4 months (IQR 22.1–32.9) with a mPFS of 28.8 months (95% CI, 22.4–37.9), significantly superior to 6.8 months (95% CI, 5.6–8.2) in the T-DM1 group [51].

In the field of GC, the DESTINY-Gastric01 trial demonstrated an ORR of 51% (95% CI 42–61%) in the T-DXd group, compared with just 14% (95% CI 6–26%) in the chemotherapy group; the median overall survival (OS) was 12.5 months (95% CI 9.6–14.3) in the T-DXd group and 8.4 months (95% CI 6.9–10.7) in the chemotherapy group [52]. Building upon this, the DESTINY-Gastric02 study, conducted by Eric Van Cutsem et al., aimed to further evaluate the efficacy and safety of T-DXd in patients with HER2-positive gastric or gastro-oesophageal junction cancer who had previously received trastuzumab therapy. The study enrolled 79 patients who received T-DXd 6.4 mg/kg intravenously every three weeks. Results demonstrated an ORR of 41.8%. Common ≥Grade 3 adverse events included anemia (13.9%) and nausea (7.6%) [53]. This persistence of activity after trastuzumab failure hinges on the unique payload mechanism of T-DXd. DXd is a topoisomerase I inhibitor, and its cytotoxic effect is completely independent of HER2 downstream signaling. Therefore, even if tumor cells acquire resistance to trastuzumab via activation of bypass pathways such as PI3K/AKT, DXd can still kill tumor cells through its independent DNA-damage mechanism. This payload-level mechanistic innovation is the core reason why T-DXd can maintain efficacy in the setting of trastuzumab resistance.

In the field of NSCLC, the Phase I study by Tsurutani et al. demonstrated that T-DXd achieved an ORR of 72.7% in patients with HER2-mutated NSCLC (*n* = 11), with a PFS of 11.3 months [54]. Subsequently, the DESTINY-Lung01 Phase II trial confirmed T-DXd’s significant efficacy in 91 HER2-mutated NSCLC patients, with an ORR of 55%, median PFS of 8.2 months, and median OS of 17.8 months. However, the incidence of ≥Grade 3 adverse events was 46%, and drug-related interstitial lung disease occurred in 26% of patients [55]. The DESTINY-Lung02 study further compared two T-DXd doses (5.4 mg/kg versus 6.4 mg/kg), yielding ORRs of 49.0% and 56.0%, respectively, suggesting superior safety at the 5.4 mg/kg dose [56].

Beyond traditional HER2-positive disease, T-DXd has also revolutionized treatment for tumors with low HER2 expression or HER2 mutations. The DESTINY-Breast04 trial demonstrated that T-DXd significantly improves the prognosis of breast cancer with low HER2 expression (defined as IHC 1+ or IHC 2+/FISH-negative): in the HR+ cohort, mPFS was 10.1 months vs. 5.4 months (HR = 0.51); in the overall population, mPFS was 9.9 months vs. 5.1 months (HR = 0.50), and median OS was 23.4 months vs. 16.8 months (HR = 0.64) [57]. Similarly, in HER2-mutated NSCLC, T-DXd demonstrated superior efficacy (objective response rates of approximately 55–57%), whereas T-DM1 achieved an objective response rate of only 20% in HER2-overexpressing NSCLC but reached 38.1% in HER2-mutated cases [58]. This highlights the unique sensitivity of HER2-mutated tumors to the T-DXd payload and to a high drug-to-antibody ratio.

T-DXd, as an innovative antibody–drug conjugate, features a novel cleavable linker that maintains plasma stability while efficiently releasing the potent topoisomerase I inhibitor DXd within the tumor microenvironment. Its high DAR enables highly loaded drug delivery, substantially addressing tumor heterogeneity. Key clinical trial data confirm that T-DXd markedly improves survival in patients with low HER2 expression and reduces the risk of disease progression compared with T-DM1.

Nevertheless, T-DXd is not without limitations. Its high DAR and the potent, membrane-permeable DXd payload raise the risk of off-target toxicities, including interstitial lung disease. In addition, T-DXd may still develop acquired resistance, such as upregulation of efflux transporters that impact topoisomerase I inhibitors. These challenges underscore the need for continued optimization of ADC design. They have spurred the development of alternatives like RC-48, aimed at achieving a more favorable balance between efficacy and tolerability.

#### 3.2.3. RC-48

To address the limitations of T-DXd, RC-48, as a new-generation HER2-targeted ADC independently developed in China, comprises the microtubule inhibitor payload MMAE linked to a HER2 antibody via a valine–citrulline spacer [59]. This cleavable linker enables rapid payload release and exhibits a bystander effect, capable of eliminating HER2-heterogeneous tumor cells. Its payload, MMAE, is delivered via antibody-targeted delivery, reducing systemic toxicity while demonstrating favorable efficacy and safety profiles in both HER2-low and HER2-overexpressing breast cancers. RC-48 binds with high affinity and specificity to HER2 on tumor cell surfaces, subsequently mediating clathrin- and caveolin-dependent endocytosis to internalize the ADC and transport it to lysosomes [60]. Within the acidic lysosomal environment, lysosomal enzymes cleave the linker, releasing the covalently attached cytotoxic MMAE. Upon release, MMAE binds to tubulin, disrupting intracellular microtubule structures, causing cell-cycle arrest at mitosis, and ultimately inducing tumor cell apoptosis. RC-48 further inhibits downstream signaling pathways activated by HER2, thereby disrupting tumor transcription, growth, and proliferation. This multi-mechanistic synergy confers RC-48 with pronounced antitumor activity in HER2-positive tumors and shows particular efficacy in tumors exhibiting low HER2 expression or heterogeneity. Pharmacodynamic studies indicate that an optimized DAR of 4.0 achieves an optimal therapeutic window. Pharmacokinetic data in rat models show DAR-dependent half-life characteristics [61].

A Phase I/II clinical trial of RC-48 in BC conducted by Jiayu et al. demonstrated significant efficacy in both HER2-positive and HER2-low-expression advanced breast cancer. At a dose of 2.0 mg/kg every two weeks, the ORR was 42.9% in HER2-positive patients and 33.3% in HER2-low patients, with an mPFS of 5.7 and 5.1 months [54], respectively. In studies focusing on HER2-positive breast cancer, RC-48 achieved an ORR of 31.4% with an mPFS of 5.8 months [62], underscoring its notable anticancer activity in HER2-positive disease and potential activity in low-HER2 expression settings. RC-48 demonstrates notable activity in HER2-low disease due to the high potency of MMAE and its bystander effect, which allows cytotoxic damage to tumor cells beyond those with high HER2 density. Based on existing clinical evidence, RC-48 in BC treatment shows superior efficacy when used in combination therapy compared with monotherapy. A multicenter real-world study enrolling 154 patients with HER2-positive and HER2-low-expressing metastatic breast cancer demonstrated that combination therapy significantly improved outcomes compared with RC-48 alone [63].

In GC, a single-arm phase II study by Peng et al. evaluated RC-48 in patients with locally advanced or metastatic HER2-overexpressing gastric/gastroesophageal junction carcinoma (IHC 2+/3+) who had received at least two prior lines of therapy. RC-48 was administered as monotherapy (2.5 mg/kg IV every two weeks), with an ORR of 24.8% and mPFS and overall survival (OS) of 4.1 months and 7.9 months [64], respectively. Notably, RC-48 also showed potential activity in HER2-low GC (IHC 1+) [65], suggesting broader applicability across patient populations.

For UC patients, a phase II study conducted by Sheng et al. demonstrated that among patients with HER2-positive locally advanced or metastatic UC who had failed at least one prior line of chemotherapy, RC-48 achieved an ORR of 51.2%, as confirmed by a blinded independent review committee. The mPFS and OS were 6.9 months and 13.9 months, respectively. The safety profile was favorable, with no Grade 4 or 5 treatment-related adverse events observed [66].

#### 3.2.4. Other ADCs in Clinical Trials in HER2

Within the evolving therapeutic landscape, researchers are broadening their horizons to drive continuous breakthroughs in core ADC technologies. In terms of efficacy, SHR-A1811 demonstrates notable activity, achieving an ORR of 61.6% across HER2-positive breast cancer, HER2-low breast cancer, urothelial carcinoma, gastric/gastroesophageal junction cancer, and NSCLC [67]. Data for SYD985 have also been reported, with a mPFS of 7.0 months (95% CI, 5.4–7.2) versus 4.9 months (95% CI, 4.0–5.5) for physician’s choice, corresponding to a HR of 0.64 (95% CI, 0.49–0.84; *p* = 0.002) [68].

**Figure 3:**
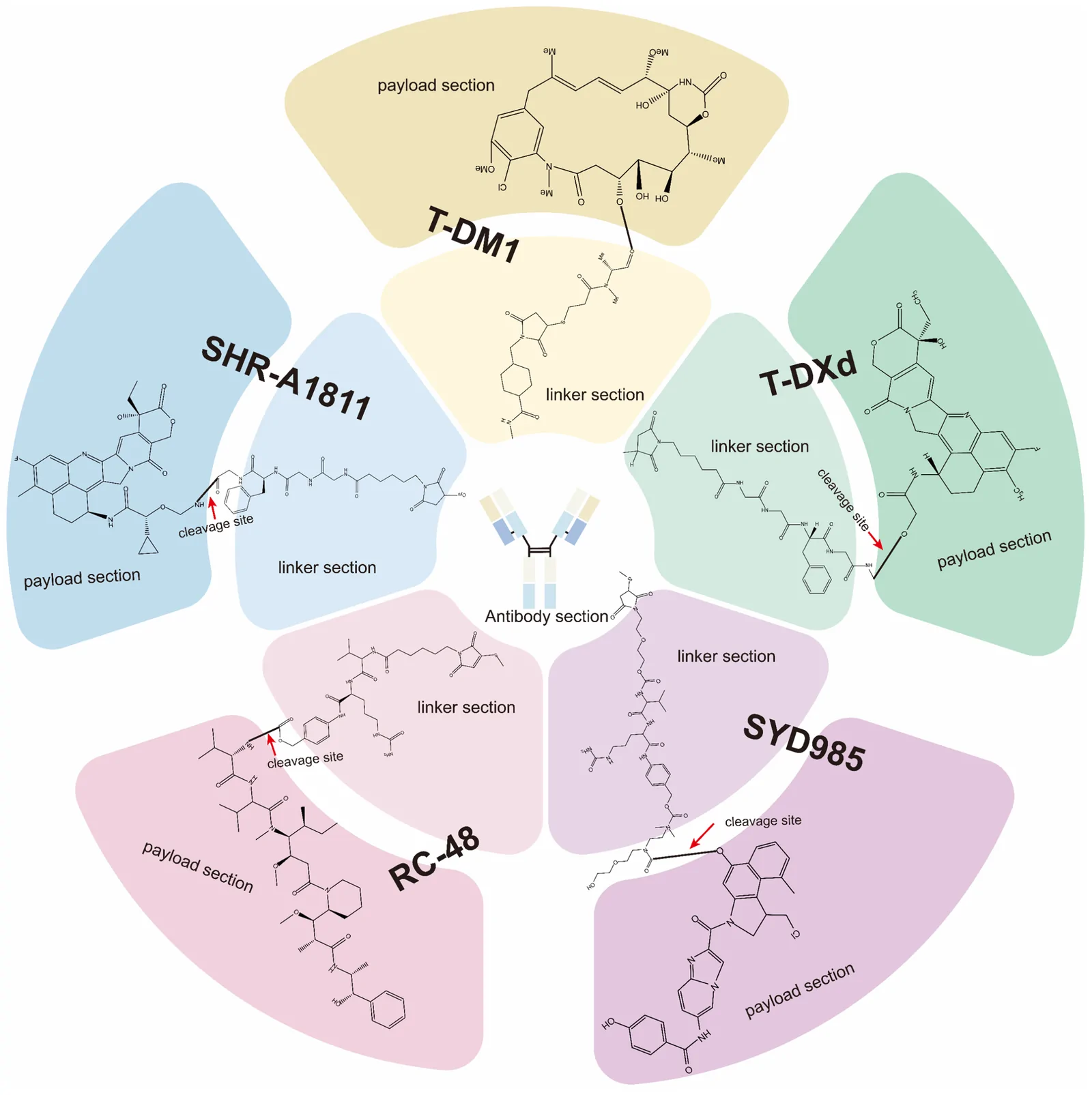
The ternary structure of HER2-targeted antibody–drug conjugates comprises a monoclonal antibody, a linker, and a cytotoxic drug.

On the safety front, distinct ADCs exhibit different safety profiles. Notably, the incidence of T-DXd-induced interstitial pneumonia has drawn attention, whereas SHR-A1811 shows a comparatively lower rate of 3.2% [67]. For SYD985, the most common adverse events were fatigue, dry eyes, conjunctivitis, and increased lacrimation, with the most frequent Grade 3/4 events being neutropenia and conjunctivitis (Figure 3).

### 3.3. Payload Cross-Resistance in HER2

The mechanisms of resistance to ADCs are closely related to the mode of action of their payloads, and understanding these relationships is crucial for optimizing sequential treatment strategies. ADCs carrying a PBD dimer payload (e.g., SG3199, which releases tesirine) display distinctive pharmacological properties: in vitro, SG3199 demonstrates picomolar cytotoxicity across diverse solid tumor and hematologic cancer cell lines (mean GI50 ≈ 151.5 pM). It induces cell death by rapidly forming interstrand DNA crosslinks that persist for more than 36 h, while displaying a very short systemic half-life (approximately 8 min) after intravenous administration in rats [69]. These characteristics render it a highly effective ADC warhead, although acquired resistance may still occur. Studies in hematologic (Karpas-299) and solid tumor (NCI-N87) cell lines show that resistance to PBD-ADCs and their free payload SG3199 can be induced by gradual, stepwise increases in concentration, with fold-resistance spanning from several-fold to several thousand-fold and exhibiting cross-resistance among distinct PBD-ADCs. Mechanistically, interstrand DNA cross-linking is diminished in resistant cells, and ATP-binding cassette (ABC) transporter genes ABCG2 and ABCC2 are significantly upregulated, while ABCB1 (MDR1) remains unaffected; inhibition or siRNA-mediated knockdown of these transporters partially restores drug sensitivity [70]. This finding suggests that drug efflux pumps constitute a key mechanism underlying acquired resistance to PBD-ADCs. In contrast, the targets and resistance pathways of microtubule inhibitors (e.g., DM1, MMAE) and topoisomerase I inhibitors (e.g., DXd, SN-38) are fundamentally different. Clinically, T-DXd (a topoisomerase I inhibitor) remains effective after resistance to T-DM1 (a microtubule inhibitor); it is precisely this mechanistic difference that prevents cross-resistance. However, for SG (SN-38) and Datopotamab deruxtecan (Dato-DXd) (DXd)—both topoisomerase I inhibitors—the theoretical risk of cross-resistance exists due to their shared target. Indeed, a subgroup analysis of a Dato-DXd study showed that the ORR was higher (44%) in patients without prior topoisomerase I inhibitor exposure than in the overall population (32%), suggesting that prior exposure may diminish the efficacy of subsequent ADCs of the same class. Against this backdrop, ADCs employing payloads with distinct mechanisms of action (e.g., RC-48 carrying the microtubule inhibitor MMAE) may provide valuable alternatives for patients who are resistant to or intolerant of topoisomerase I inhibitor ADCs, potentially overcoming resistance by bypassing common resistance pathways (such as efflux-pump upregulation). The transporter-mediated mechanisms described in the aforementioned PBD-ADC resistance studies further emphasize the importance of payload-specific resistance, providing a theoretical basis for avoiding cross-resistance in sequential clinical therapy.

**Table 1:** Systematic comparison of core characteristics among different anti-HER2 antibody–drug conjugates through detailed analysis.

Drug Name	Company	Target	Antibody	Linker	Payload	DAR	Clinical Stage	Most Common/Notable Adverse Events	Year	References
T-DM1	Genentech	HER2	Trastuzumab	Non-cleavable maleimidomethylene cyclohexane-1-carboxylic acid (MCC) thioether linker arm	Maytansinoid derivative, DM1	3.5	Approved for marketing by the FDA	Thrombocytopenia, elevated liver enzymes, interstitial pneumonia, cardiotoxicity	2013	[70]
T-DXd	AstraZeneca and Daiichi Sankyo are jointly developing and commercializing	HER2	Trastuzumab	Cleavable GGFG linker	Topoisomerase I Inhibitors, DXd	7–8	Approved for marketing by the FDA	Interstitial lung disease, neutropenia and left ventricular dysfunction	2019	[40]
RC-48	RemeGen Rongchang Biopharmaceutical (Yantai) Co., Ltd.	HER2	Hertuzumab	Cleavable vc-PABC linker	MMAE	4	Approved for marketing in China	Peripheral sensory neuropathy, leukopenia, increased AST, and neutropenia	2021	[71,72]
SHR-A1811	Hengrui Pharmaceuticals	HER2	Trastuzumab	Enzyme-cleavable linker containing malonylimidazole tetrapeptide (GGFG)	Novel Topoisomerase I Inhibitor Payload SHR9265	5.7	Phase I clinical trial	Interstitial lung disease, diarrhea	2025	[73]
SYD985	Synthon Biopharmaceuticals BV	HER2	trastuzumab duocarmazine	Peptide linker cleavable by cathepsin B (containing the valine-citrulline sequence)	Seco-DUBA, a derivative of duocarmycin	2	3:1 (NCT03262935)	Conjunctivitis, stomatitis, fatigue and loss of appetite	2019	[74]

## 4. The Biological Characteristics of Trop-2 and Its Role in Cancer

### 4.1. Biological Properties of Trop-2

Trop-2 (TACSTD2) is a transmembrane glycoprotein involved in calcium signal transduction. It is abnormally overexpressed in various tumor types [75]. The Trop-2 gene is localized to the 1p32 region of chromosome 1 [72]. Its protein product comprises 323 amino acids, including a hydrophobic leader peptide, an extracellular domain, a transmembrane domain, and a cytoplasmic tail. The extracellular domain contains an epidermal growth factor-like repeat sequence (EGF-like domain), a cysteine-rich domain, and a thyroglobulin type-1 domain. This multi-domain architecture enables Trop-2 to mediate cell–cell interactions and signal transduction [74]. Its cytoplasmic tail contains a highly conserved phosphatidylinositol 4,5-bisphosphate binding sequence, which overlaps with the phosphorylation site for protein kinase C. The cytoplasmic tail contains a conserved PIP2 binding site and PKC phosphorylation site, regulating its signal transduction [76].

Trop-2 expression is relatively low in normal tissues but is significantly upregulated in numerous cancers, making it an ideal target for therapeutic intervention. Overexpressed Trop-2 drives tumor growth by activating intracellular calcium signaling, the MAPK pathway, and cyclin D/E [77]. Trop-2 also activates the AKT pathway to promote cell cycle progression and inhibit apoptosis [78]. Furthermore, Trop-2 expression is regulated by epithelial–mesenchymal transition (EMT) processes, wherein EMT transcription factors suppress Trop-2 expression, resulting in heterogeneous expression patterns during tumor progression and metastasis. This heterogeneity correlates strongly with cancer aggressiveness and poor prognosis [75]. Particularly in BC, high Trop-2 expression shows strong correlation in triple-negative BC, with its overexpression significantly associated with increased distant metastasis rates and reduced patient survival. This finding offers novel insights and directions for clinical treatment.

Trop-2 has been established as a prognostic marker in multiple tumors [79]. Trop-2 overexpression correlates with reduced T-cell infiltration and immune evasion (possibly via downregulation of T-cell chemokines), providing a theoretical basis for ADC development [80]. This characteristic provides a theoretical basis for developing ADCs targeting Trop-2. Research indicates that inhibiting Trop-2 expression or employing monoclonal antibodies targeting Trop-2 significantly enhances therapeutic outcomes in certain malignancies. This is achieved through mechanisms such as blocking the Trop-2-mediated AKT signaling axis and/or inducing DNA damage and apoptosis [78].

Targeting Trop-2 has become a significant direction in oncology drug development. To date, targeted therapeutics and ADCs have been successfully developed against Trop-2. These targeted agents precisely bind to Trop-2, blocking its interaction with downstream signaling molecules and thereby inhibiting tumor cell growth signaling pathways. Gaining deeper insights into Trop-2’s function and regulatory mechanisms may yield novel strategies and approaches for cancer treatment.

### 4.2. Applications of Trop-2-Targeting ADCs in Specific Cancers

The high expression of Trop-2 across multiple cancer types positions it as a promising universal therapeutic target for cross-cancer treatment, broadening therapeutic applicability. In novel drug development, deepening understanding of Trop-2’s mechanism of action holds promise for creating more highly effective, low-toxicity targeted therapies and ADCs, thereby further enhancing treatment efficacy. Current ADCs targeting Trop-2 primarily include SG, Dato-DXd, Sacituzumab tirumotecan (SKB264), ESG-401, JS-108, and FDA018 [35].

#### 4.2.1. SG

SG is an ADC formed by conjugating the humanized monoclonal antibody hRS7 IgG1κ against Trop-2 with SN-38 via the cleavable linker CL2A. SN-38 is the active metabolite of the topoisomerase I inhibitor irinotecan. This ADC is characterized by a high DAR, wherein each antibody binds 7.5–8 SN-38 molecules via the unique, hydrolysable proprietary linker CL2A. This design leverages SN-38’s significantly improved toxicity profile compared to its parent drug irinotecan [81]. In 2019, SG received FDA approval as the first non-HER2-targeted ADC for unresectable locally advanced or metastatic TNBC in patients who had received two or more prior systemic therapies. This landmark approval signaled a new era for ADCs in TNBC treatment and laid the foundation for further research and applications [82]. Research indicates that SG primarily exerts its effects by inhibiting tumor cell proliferation and migration through suppression of the epidermal growth factor receptor and its downstream PI3K-AKT signaling pathway. The excessive activation of the epidermal growth factor receptor is closely associated with the development and progression of various tumors, and SG’s mechanism of action involves downregulating this signaling pathway, thereby limiting the proliferative capacity of tumor cells [83]. SG exerts cytotoxic effects through its conjugated chemotherapeutic agent SN-38, demonstrating significant efficacy in clinical trials. Particularly in patients with recurrent or refractory TNBC following chemotherapy, SG has markedly improved both PFS and OS. Unlike conventional chemotherapeutic agents such as irinotecan, ADCs deliver payloads to tumor cells with high specificity via antibody–antigen-mediated binding, markedly increasing drug concentration at the target site while reducing systemic exposure, thereby preserving or enhancing antitumor activity while minimizing toxicity.

Currently, the approved indications for SG in BC are well defined: TNBC received approval in 2019 based on the ASCENT trial, and hormone receptor-positive/HER2-negative BC (HR+/HER2− BC) received approval in 2023 based on the TROPiCS-02 trial. The pivotal Phase III trials underpinning SG’s efficacy are ASCENT and TROPiCS-02. In ASCENT, SG significantly improved outcomes versus chemotherapy, with a mPFS of 5.6 vs. 1.7 months (HR = 0.41) and median overall survival (mOS) of 12.1 vs. 6.7 months (HR = 0.48 [84]. Prominent activity in triple-negative breast cancer is mediated by high Trop-2 expression and potent inhibition of topoisomerase I by SN-38, exploiting the intrinsic DNA repair defects characteristic of TNBC. In TROPiCS-02, SG again demonstrated a survival advantage, significantly prolonging median PFS (5.5 vs. 4.0 months; HR = 0.66) and median OS (14.4 vs. 11.2 months; HR = 0.79) compared with chemotherapy, marking the first Trop-2–ADC to show a survival benefit in HR+ BC [84].

For NSCLC, the IMMU-132–01 Phase I/II trial by Bardia et al. evaluated SG in 495 patients with refractory metastatic epithelial carcinoma, including 54 NSCLC patients. The ORR was 16.7%, with a duration of response of 6 months, OS of 7.3 months, and mPFS of 4.4 months [85].

In UC, the Phase I/II IMMU-132-01 basket trial evaluated SG in patients with advanced solid tumors; within the metastatic UC cohort (45 patients), the ORR was 31%. In the Phase II TROPHY-U-01 study (NCT03547973), 113 patients with metastatic UC who had previously received platinum-based chemotherapy and immunotherapy were treated with SG, achieving an ORR of 27.4% with a median duration of response of 7.2 months [86].

SG employs the antibody hRS7, targeting the Trop-2 antigen, to efficiently deliver the topoisomerase I inhibitor SN-38 to tumor cells via a cleavable linker (CL2A), thereby addressing tumor heterogeneity. Collectively, these studies support SG’s therapeutic potential across BC, NSCLC, and UC, with demonstrated improvements in survival outcomes and ORRs across multiple cancer types and subtypes. The success of SG is largely demonstrated in patients with advanced TNBC and HR+/HER2- BC who have received multiple lines of therapy (chemotherapy and/or immunotherapy). At present, evidence for SG in the first-line setting is insufficient, and its standard positioning remains as a later-line option following failure of prior therapies.

SG design embodies the challenge of balancing efficacy and toxicity in ADCs. Its high drug-to-antibody ratio (DAR) and a cleavable linker can boost anti-tumor activity via the bystander effect. However, the linker’s poor plasma stability can drive substantial hematologic and gastrointestinal toxicities. To address this limitation, the next-generation TROP2-targeting ADC Dato-DXd uses a plasma-stable, selectively cleavable linker, which effectively reduces off-target toxicity. Its controllable safety supports sequential use in late-stage tumors, while preserving the bystander effect and markedly lowering systemic toxicity. This provides a more favorable strategy for clinical management.

#### 4.2.2. Dato-DXd

The core of this concept is the unique structural design of Dato-DXd. Unlike SG, which has a high drug-to-antibody ratio (DAR) of about 7.6, Dato-DXd uses a lower DAR of 4:1. It also employs a plasma-stable tetrapeptide linker to deliver the DXd payload effectively. This design aims to minimize hematologic and gastrointestinal toxicities that limit SG’s clinical use.

In contrast to SG, Dato-DXd (Datroway) has been approved by the FDA for HR+/HER2− breast cancer based on the pivotal Phase III TROPION-Breast01 trial. It is designed to achieve a wider therapeutic window by improving safety. A key advantage of Dato-DXd lies in its distinct design: whereas SG employs a high drug-to-antibody ratio (DAR) of approximately 7.6, Dato-DXd uses a lower DAR of 4:1 and incorporates a tetrapeptide-cleavable linker to deliver the DXd payload. This design is intended to reduce hematologic toxicities (e.g., neutropenia) and gastrointestinal toxicities (e.g., diarrhea), which are among the most common adverse events associated with SG [87]. By binding to Trop-2, Dato-DXd activates multiple signaling pathways, including PI3K/AKT and MAPK, which are crucial for cell growth and survival. By modulating these pathways, Dato-DXd may promote tumor cell apoptosis and potentially remodel the tumor microenvironment, thereby enhancing the host anti-tumor immune response (Table 2) [87]. Dato-DXd demonstrated in vivo antitumor activity in Trop-2-expressing xenograft models, with accumulated DXd inducing DNA damage—an effect not observed with isotype-control IgG-ADCs or anti-Trop-2 antibodies. Dato-DXd also demonstrated potent antitumor activity and tumor regression across multiple Trop-2-expressing xenografts. In the Phase III TROPION-Breast01 study in HR+/HER2− breast cancer, Dato-DXd demonstrated a statistically significant and clinically meaningful improvement in median progression-free survival (6.9 months) versus 4.9 months for investigator’s choice of chemotherapy (HR = 0.63, 95% CI 0.52–0.76, *p* < 0.0001) [88]. The relatively low DAR and the tetrapeptide linker reduce off-target toxicity, enabling prolonged dosing; in HR-positive breast cancer, this facilitates delivery of sustained DNA damage to tumors that progressed after endocrine therapy and retain intact DNA repair pathways, thereby explaining the coexistence of progression-free survival benefit and favorable safety. Beyond efficacy, Dato-DXd showed a favorable safety profile with a significantly lower incidence of grade ≥ 3 treatment-related adverse events compared with investigator’s choice of chemotherapy (20.8% vs. 44.7%), which may offer a meaningful clinical advantage for long-term therapy or patients with poor performance status [89].

A study involving 85 patients with HR+/HER2− BC (41 cases) and TNBC (44 cases) demonstrated significant antitumor activity for Dato-DXd: the HR+/HER2− BC group achieved an ORR of 26.8% and median PFS of 8.3 months, while the TNBC group achieved an ORR of 31.8%, a median duration of response of 16.8 months, and a median PFS of 4.4 months. Sustained responses were observed across all subgroups. Stomatitis occurred in 82.9% of HR+/HER2− BC patients and 72.7% of TNBC patients, supporting the drug’s potential efficacy and safety profile [90].

In the NSCLC cohort of the TROPION-PanTumor01 trial, 180 patients were enrolled across dose levels of 4–8 mg/kg. The ORRs were 22% (4 mg/kg), 26% (6 mg/kg), and 23.8% (8 mg/kg). In the 6 mg/kg cohort, the median PFS was 6.9 months and median OS was 11.4 months [91].

ADCs targeting Trop-2 represent a significant advance in precision oncology and offer novel therapeutic options for a range of malignant tumors. Current clinical trials have demonstrated meaningful efficacy across multiple tumor types, including activity in drug-resistant and metastatic disease. Trop-2 is aberrantly overexpressed in a substantial proportion of epithelial tumors, making it an attractive therapeutic target. Several Trop-2-directed ADCs have been shown to prolong progression-free survival and improve objective response rates. As research progresses, these agents are expected to transform current treatment paradigms and address the limitations of conventional therapies.

#### 4.2.3. Other ADCs in Clinical Trials in Trop-2

**Figure 4:**
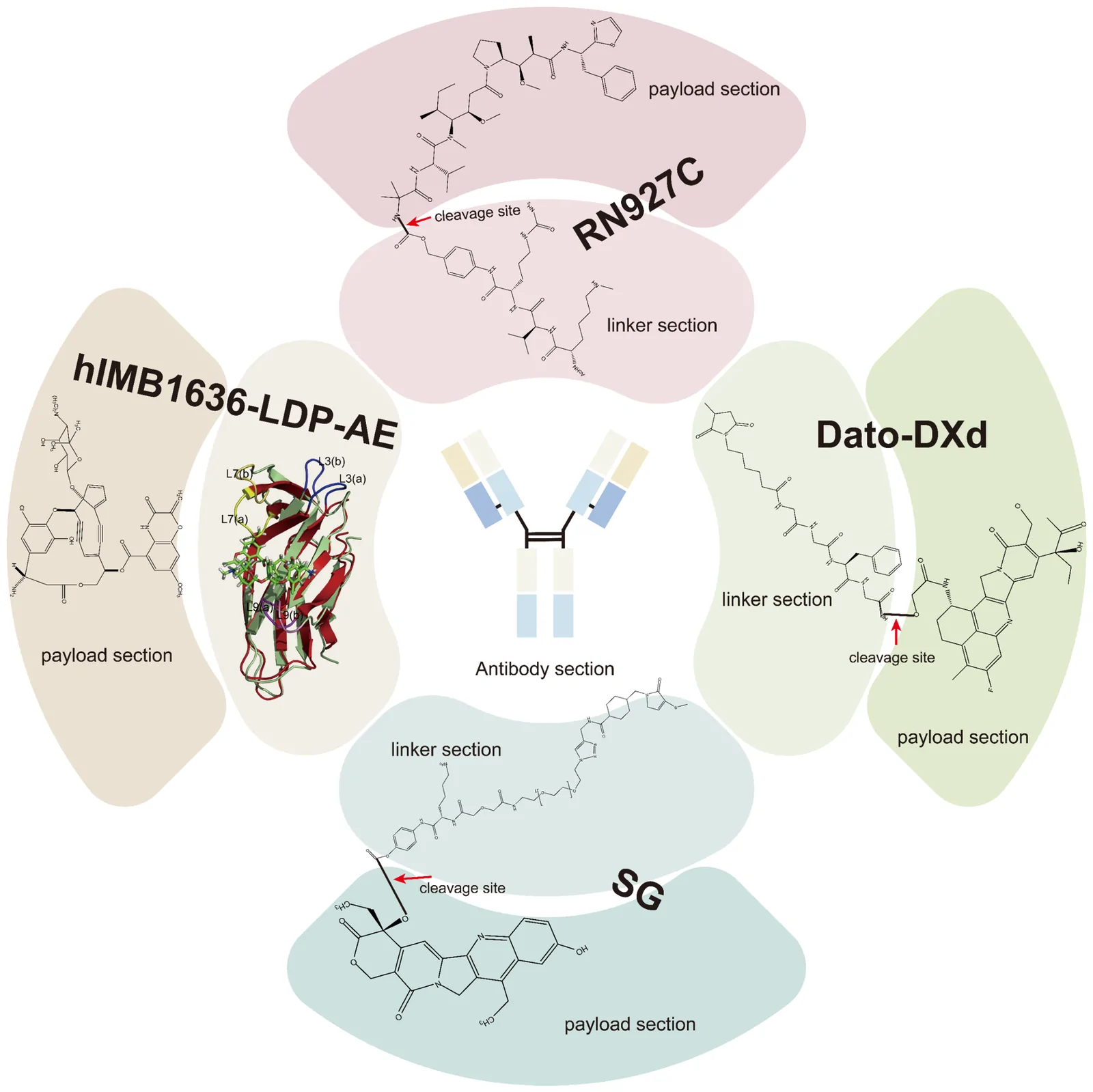
The ternary structure of Trop-2-targeted antibody–drug conjugates comprises a monoclonal antibody, a linker, and a cytotoxic drug [97].

Novel Trop-2-targeted ADCs demonstrate significant potential in the field of antitumor therapy, with hIMB1636-LDP-AE exhibiting particularly outstanding efficacy. In vitro experiments reveal that this compound significantly inhibits the proliferation, migration, and tumor spheroid formation of both lung cancer and BC cells. Animal studies further validate its potent antitumor effects: at a dose of 0.8 mg/kg, hIMB1636-LDP-AE achieved an inhibition rate of 76.62 ± 10.99% against HCC827 tumors, while exhibiting inhibition rates of 56.15 ± 10.85%, 77.59 ± 1.78%, and 67.94 ± 6.91% against H1975, MDA-MB-468, and MCF-7 tumors, respectively. No significant toxic side effects were observed throughout the experimental process (Table 2) [92]. Another Trop-2-targeted ADC, RN927C, also demonstrated superior efficacy in TNBC treatment. In the TNBC PDX model CTG-1017, a single injection of RN927C induced regression of large tumors measuring approximately 830 mm in diameter, with regression persisting for over 60 days [93]. Notably, when administered again to tumors regrowing 63 days after the initial injection, RN927C still achieved prolonged tumor regression (Figure 4) [94].

### 4.3. Payload Cross-Resistance in Trop-2

Regarding the issue of payload cross-resistance in Trop-2-targeted ADCs, the choice of subsequent treatment strategies is determined by the payload’s mechanism of action. Trop-2 ADCs currently in clinical development primarily incorporate topoisomerase I inhibitors (such as SG’s SN-38 and Dato-DXd’s DXd) or microtubule inhibitors (such as RN927C’s PF-06380101). Since both SN-38 and DXd target topoisomerase I, there is a theoretical risk of cross-resistance. A subgroup analysis of a Dato-DXd study showed that the objective response rate (44%) among patients who had not previously received topoisomerase I inhibitor therapy was higher than that of the overall population (32%), suggesting that prior exposure to drugs of the same class may diminish subsequent efficacy. In contrast, ADCs carrying microtubule inhibitors (such as RN927C) inhibit cell division by binding to tubulin; their mechanism of resistance is fundamentally different from that of topoisomerase I inhibitors. Studies have confirmed that RN927C demonstrates superior efficacy against tumors resistant to paclitaxel or gemcitabine in various xenograft models, including pancreatic cancer, lung cancer, ovarian cancer, and triple-negative breast cancer, indicating that microtubule inhibitor-based ADCs can serve as an effective alternative following resistance to topoisomerase I inhibitors [93]. Furthermore, Trop-2 expression is regulated epigenetically, such as through DNA methylation, and while its expression level is correlated with ADC efficacy, it is not the sole determining factor [95]. It is worth noting that the efficacy of Trop-2 ADCs across different tumor types is also influenced by the immunological status of the tumor microenvironment; for example, tumors with high TACSTD2 expression are often accompanied by stronger immune cell infiltration, which offers potential for combination immunotherapy but may also affect ADC persistence through immune evasion mechanisms [96]. In clinical practice, when patients develop resistance to SG or Dato-DXd, switching to an ADC carrying a microtubule inhibitor (such as an RN927C analog) may circumvent the common efflux resistance mechanism caused by the upregulation of ATP-binding cassette transporters (e.g., ABCB1, ABCG2), although this hypothesis requires further validation with clinical data in the field of Trop-2 ADCs. Therefore, sequential treatment strategies based on differences in payload mechanisms hold promise for overcoming cross-resistance and prolonging patient benefit.

**Table 2:** Systematic comparison of core characteristics among different anti-trop-2 antibody–drug conjugates through detailed analysis.

Drug Name	Company	Target	Antibody	Linker	Payload	DAR	Clinical Stage	Most Common/Notable Adverse Events	Year	References
SG	Gilead Sciences (following its acquisition of Immunomedics)	Trop-2	hRS7 IgG1κ	Cleavable Linker CL2A	SN-38	7.6	Approved for marketing by the FDA	Myelosuppression and diarrhea	2023	[98]
Dato-DXd	Daiichi Sankyo, in collaboration with AstraZeneca (Daiichi Sankyo holds exclusive rights in Japan and granted AstraZeneca co-development rights in other markets in 2020)	Trop-2	Humanized anti-Trop-2 IgG1 monoclonal antibody	Tetapeptide cleavable linker	DXd (Isatriptan derivative, a DNA topoisomerase I inhibitor)	4	Phase III Clinical Trial	Interstitial lung disease/pneumonitis, infusion-related reactions, oral mucositis/stomatitis, and ocular surface events	2021	[99,100]
hIMB1636-LDP-AE	Institute of Medical Biotechnology, Chinese Academy of Medical Sciences	Trop-2	hIMB1636	Non-cleavable peptide linker (SGGPEGGS)	Alkenyl-diyne chromophore (AE, derived from lidaomycin LDM)	2	Preclinical research phase	Preclinical research phase	2024	[92]
RN927C	Pfizer/Rinat	Trop-2	Humanized Anti-Trop-2 hIgG1 Antibody	AcLys-VC-PABC	PF-06380101 (Potent antimitotic agent that inhibits tubulin polymerization)	1.9	Preclinical research phase	Preclinical research phase	2016	[93]

## 5. Advances in Resistance Management, Combination Strategies, and Biomarkers in ADCs Therapy

### 5.1. Mechanisms of ADCs Resistance and Precision Drug Switching Based on Payload-Mediated Cross-Resistance

In terms of mechanisms and optimal sequencing, cross-resistance among payloads is a clinically important phenomenon [101]. Once tumor cells acquire resistance to ADCs carrying topoisomerase I inhibitors (e.g., T-DXd or SG), subsequent treatment options may be limited [102]. A study by Rampa et al. showed that resistance in T-DXd- and SG-resistant breast cancer cells is primarily driven by upregulation of efflux transporters (e.g., ABC transporters), with little loss of antigen expression; switching to ADCs carrying microtubule inhibitors (e.g., MMAE or DM1) can restore antitumor activity, suggesting that payload diversification may be an effective strategy to overcome cross-resistance [103]. Additionally, changes in antigen expression represent another key mechanism: after ADC treatment, surface expression of HER2 or Trop-2 may be downregulated or lost, which helps explain why sequential use of ADCs targeting the same antigen is often ineffective [104]. For example, after treatment with HER2-directed ADCs, polyploid giant cancer cells (PGCCs) showed decreased HER2 expression but upregulated nectin-4, providing evidence for target switching [4]. Intracellular abnormalities, including impaired endocytosis, lysosomal dysfunction, and ABC transporter-mediated drug efflux, also contribute to the development of drug resistance [105]. Based on these mechanisms, sequential treatment strategies should rely on dynamic biomarker monitoring and incorporate an understanding of payload-cross-resistance to design personalized treatment sequences. For example, in HER2-positive breast cancer, if HER2 remains highly expressed after progression on T-DXd, T-DM1 may be attempted; if expression has shifted to low levels, Trop-2 ADCs such as SG or Dato-DXd may be considered, or RC-48 may be used to achieve a dual switch in both target and payload [106].

Payload cross-resistance has important clinical implications for therapy sequencing. After T-DXd, subsequent ADCs with the same payload mechanism are unlikely to provide meaningful benefit; sequencing should be guided by dynamic biomarker monitoring, with HER2 status informing whether to switch payload mechanisms or pivot to Trop-2-targeted or other antigen-targeted ADCs. Prospective trials are needed to validate these sequencing strategies.

### 5.2. ADCs Combination Therapies: Immunotherapy, Targeted Therapy, Synergistic Strategies, and Clinical Applications

**Figure 5:**
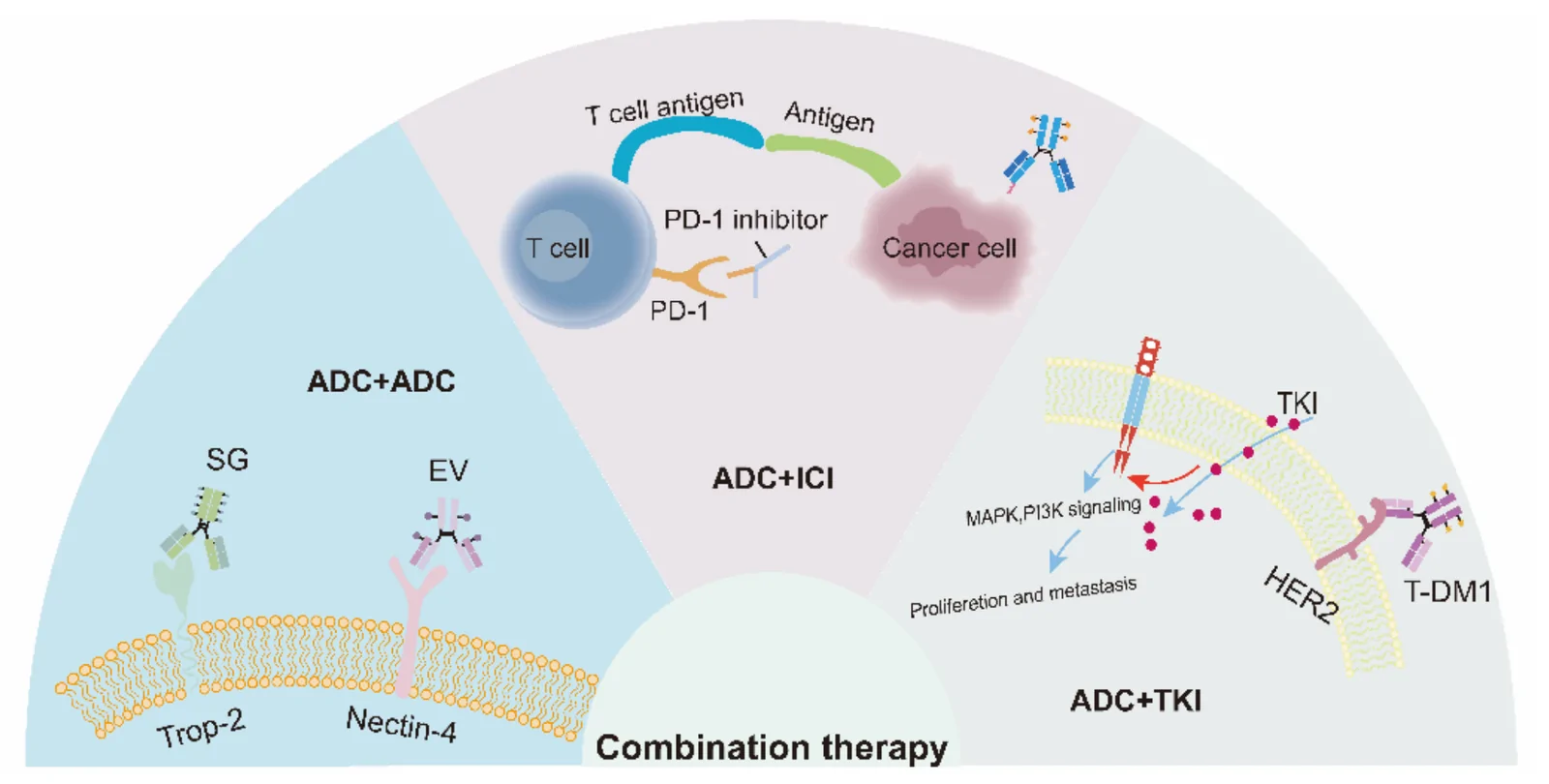
Synergistic therapeutic strategies for antibody–drug conjugates primarily encompass three types: combination with other antibody–drug conjugates, combination with immune checkpoint inhibitors, and combination with tyrosine kinase inhibitors.

In combination therapy, the synergistic mechanism between ADCs and immune checkpoint inhibitors is primarily mediated by ADC-induced immunogenic cell death (ICD). When ADCs kill tumor cells, they release antigens and damage-associated molecular patterns (DAMPs), which enhance tumor immunogenicity and improve the efficacy of immune checkpoint inhibitors [4]. Several reviews emphasize that combining ADCs with immunotherapy can substantially enhance antitumor efficacy via synergistic mechanisms, with synergistic effects observed in both preclinical and clinical studies. For example, in BC, a clinical trial evaluating T-DM1 in combination with pembrolizumab in 20 patients with metastatic HER2-positive BC reported an ORR of 20% (95% CI 5.7–43.7%), an mPFS of 9.6 months (95% CI 2.8–16.0 months), and a manageable safety profile [107]. In NSCLC, the Phase I/II trial TROPION-LUNG02 evaluated Dato-DXd in combination with pembrolizumab (with or without platinum-based chemotherapy) in patients with advanced or metastatic disease, achieving an ORR of 60% (95% CI 36–81) and a median PFS of 8.3 months (95% CI 6.8–11.8) with manageable safety. This remains the largest study to date evaluating the combination of an ADC with an anti-PD-1/PD-L1 agent in first-line NSCLC (Figure 5) [108]. In addition, combinations of ADCs with other ADCs have shown promise; for example, preliminary efficacy has been observed for SG in combination with enfortumab vedotin in urothelial cancer. Among metastatic urothelial carcinoma (mUC) patients who progressed after platinum-based chemotherapy or immunotherapy, the ORR reached up to 70% with SG plus enfortumab vedotin [109]. The combination of ADCs with tyrosine kinase inhibitors has also demonstrated synergistic effects; for instance, in HER2-positive breast cancer, T-DM1 plus lapatinib yielded an ORR of 63%, significantly higher than monotherapy [106]. However, combination therapy requires careful consideration of additive toxicity risks, particularly interstitial pneumonia, peripheral neuropathy, and gastrointestinal toxicities, which must be managed via optimized dosing sequences, dose adjustments, and proactive supportive care. For example, cytotoxic ADC payloads may induce characteristic adverse reactions such as MMAE-associated peripheral neuropathy and topoisomerase inhibitor-related gastrointestinal toxicity. Combination therapy may also exacerbate immune-related adverse events or typical toxicities of TKIs (e.g., diarrhea and skin reactions), which must be managed through optimized dosing sequences, dose adjustments, and supportive care [92]. With the emergence of novel ADCs and combination regimens, ADC-based combination therapy is expected to further expand its scope of application, contributing to more precise and personalized cancer treatment. Current research actively explores predictive biomarkers—such as tumor antigen expression levels and features of the immune microenvironment—to optimize patient selection. As new regimens integrating ADCs, immunotherapy, and targeted therapy emerge, ADC-based combination therapy is expected to broaden its applications and advance precision oncology.

### 5.3. Biomarker Testing and Personalized Treatment

Traditional HER2 testing methods (immunohistochemistry and fluorescence in situ hybridization) have limitations, including semi-quantitative assessment, substantial subjectivity, and low throughput, which hinder accurate detection of low-level HER2 expression [110]. There is an imperative to develop high-throughput, more precise methods, including next-generation sequencing (NGS) for HER2 mutations to identify NSCLC patient subgroups likely to benefit from targeted therapy, liquid biopsy for HER2 amplification or mutations in circulating tumor DNA (ctDNA) to enable dynamic monitoring, and digital pathology or AI-assisted IHC scoring to standardize interpretation of low-level HER2 expression [105]. The absence of standardized and clinically validated Trop-2 diagnostic assays represents a major bottleneck for the precise application of Trop-2-targeted ADCs. Unlike HER2, which benefits from a mature IHC scoring system, Trop-2 expression is commonly assessed with nonstandardized IHC approaches, using different antibodies and variable scoring thresholds. Currently, patient selection largely relies on histologic subtype, which is an imperfect surrogate. Developing a reproducible, clinically validated Trop-2 detection method-potentially integrated with additional biomarkers-will be critical for identifying patients most likely to benefit and for understanding the mechanisms underlying primary resistance to these therapies. Developing reliable and reproducible testing methods and establishing clinically meaningful benefit thresholds are among the most urgent clinical needs in this field. Furthermore, integrated multi-omics analyses (e.g., whole-exome sequencing, transcriptomics, and proteomics) can deepen our understanding of resistance mechanisms and reveal novel predictive biomarkers, thereby optimizing patient selection and treatment sequencing. With advances in these biomarker technologies, future precision sequential and combination ADC therapies will become more personalized, offering the potential to overcome drug resistance and improve patient outcomes.

## 6. Discussion

ADCs targeting HER2 and Trop-2 have demonstrated remarkable clinical efficacy, significantly improving outcomes across various solid tumors. Their innovative structure, combining precise antibody targeting, stable linkers, and potent payloads, confers unique pharmacodynamic and pharmacokinetic properties. This enables tumor-specific accumulation via antigen-mediated endocytosis and broadens cell killing through cleavable-linker-regulated bystander effects. These pharmacokinetic advantages, characterized by high tumor selectivity and prolonged half-life, ultimately overcome the off-target toxicity limitations of conventional chemotherapy.

T-DM1 and T-DXd, both targeting HER2, have significantly improved survival for HER2-positive and HER2-low patients. However, this precision is challenged by potential cross-resistance between payloads, highlighting a core tension: T-DXd, with its more efficient payload, further extends treatment to populations resistant to T-DM1, exemplifying this evolving dynamic.

As a broadly expressed target, Trop-2 also presents a challenge: maximizing while managing, particularly in normal tissues with low Trop-2 expression. Trop-2-targeted SG and Dato-DXd have overcome key bottlenecks in TNBC and HR+/HER2-low BC, with survival benefits confirmed across multiple studies. Further research into these subtypes may yield additional novel ADCs, potentially reshaping current paradigms.

In the future, we must overcome the limitations of single-antigen approaches. Research should integrate antigen expression patterns, drug delivery mechanisms, and tumor biological characteristics to enable the design of personalized ADCs and the application of combination therapies, guided by evidence-based medicine. It is crucial to investigate the synergistic effects of ADCs and other treatments, such as immune checkpoint inhibitors. Developing appropriate combination therapy regimens is key to overcoming existing drug resistance and addressing the issue of drug toxicity.

Future ADC technology will evolve toward multifunctionality, including bispecific ADCs and ADCs carrying multiple payloads. Bispecific ADCs can simultaneously recognize two different antigens, thereby enhancing tumor selectivity and reducing off-target toxicity, while dual-payload ADCs (such as those containing both a topoisomerase I inhibitor and a microtubule inhibitor) may overcome drug resistance through synergistic mechanisms. These new designs are expected to further broaden the therapeutic window of ADCs.

In summary, ADCs targeting HER2 and Trop-2 offer renewed hope for the treatment of solid tumors. Whilst current research findings are encouraging, further exploration of their potential remains necessary, alongside the integration of multiple therapeutic approaches to optimize treatment outcomes. At the same time, intensifying research on safety and tolerability—while striking a balance between enhancing efficacy and managing toxicity—remains key to achieving widespread clinical application of this technology. To ultimately provide patients with more comprehensive and effective treatment options, we must prioritize personalized therapy and proactively address these inherent trade-offs.

## Data Availability

No new data were created or analyzed in this study. Data sharing is not applicable to this article.

## References

[1] Tarantino P., Carmagnani Pestana R., Corti C., Modi S., Bardia A., Tolaney S.M., Cortes J., Soria J.-C., Curigliano G. (2022). Antibody-drug conjugates: Smart chemotherapy delivery across tumor histologies. CA Cancer J. Clin..

[2] Hong X., Chen X., Wang H., Xu Q., Xiao K., Zhang Y., Chi Z., Liu Y., Liu G., Li H. (2023). A HER2-targeted antibody-drug conjugate, RC48-ADC, exerted promising antitumor efficacy and safety with intravesical instillation in preclinical models of bladder cancer. Adv. Sci..

[3] Udofa E., Sankholkar D., Mitragotri S., Zhao Z. (2024). Antibody drug conjugates in the clinic. Bioeng. Transl. Med..

[4] Li S., Zhao X., Fu K., Zhu S., Pan C., Yang C., Wang F., To K.K.W., Fu L. (2025). Resistance to antibody-drug conjugates: A review. Acta Pharm. Sin. B.

[5] Ehrlich P. (1913). Address in pathology, on chemotherapy: Delivered before the Seventeenth International Congress of Medicine. BMJ.

[6] Asmani A.Z.A., Zainuddin A.F.F., Azmi Murad N.A., Mohd Darwis N.H., Suhaimi N.S., Zaini E., Taher M., Susanti D., Khotib J. (2024). Immunogenicity of monoclonal antibody: Causes, consequences, and control strategies. Pathol. Res. Pract..

[7] Drago J.Z., Modi S., Chandarlapaty S. (2021). Unlocking the potential of antibody-drug conjugates for cancer therapy. Nat. Rev. Clin. Oncol..

[8] Gröger M., Fischer G.F., Wolff K., Petzelbauer P. (1999). Immune complexes from vasculitis patients bind to endothelial Fc receptors independent of the allelic polymorphism of FcgammaRIIa. J. Investig. Dermatol..

[9] Kaplan M., Na’amad M., Kenan A., Rudensky B., Hammerman C., Vreman H.J., Wong R.J., Stevenson D.K. (2009). Failure to predict hemolysis and hyperbilirubinemia by IgG subclass in blood group A or B infants born to group O mothers. Pediatrics.

[10] Giugliano F., Corti C., Tarantino P., Michelini F., Curigliano G. (2022). Bystander effect of antibody-drug conjugates: Fact or fiction?. Curr. Oncol. Rep..

[11] Yin O., Iwata H., Lin C.C., Tamura K., Watanabe J., Wada R., Kastrissios H., AbuTarif M., Garimella T., Lee C. (2021). Exposure-Response Relationships in Patients with HER2-Positive Metastatic Breast Cancer and Other Solid Tumors Treated with Trastuzumab Deruxtecan. Clin. Pharmacol. Ther..

[12] Kopp A., Hofsess S., Cardillo T.M., Govindan S.V., Donnell J., Thurber G.M. (2023). Antibody-Drug Conjugate Sacituzumab Govitecan Drives Efficient Tissue Penetration and Rapid Intracellular Drug Release. Mol. Cancer Ther..

[13] Conilh L., Sadilkova L., Viricel W., Dumontet C. (2023). Payload diversification: A key step in the development of antibody-drug conjugates. J. Hematol. Oncol..

[14] Jhaveri K., Marmé F. (2024). Current and emerging treatment approaches for hormone receptor-positive/human epidermal growth factor receptor 2-negative metastatic breast cancer. Cancer Treat. Rev..

[15] Tsuchikama K., Anami Y., Ha S.Y.Y., Yamazaki C.M. (2024). Exploring the next generation of antibody-drug conjugates. Nat. Rev. Clin. Oncol..

[16] Lu J., Jiang F., Lu A., Zhang G. (2016). Linkers having a crucial role in antibody-drug conjugates. Int. J. Mol. Sci..

[17] Adams E., Wildiers H., Neven P., Punie K. (2021). Sacituzumab govitecan and trastuzumab deruxtecan: Two new antibody-drug conjugates in the breast cancer treatment landscape. ESMO Open.

[18] Schmitt S., Machui P., Mai I., Herterich S., Wunder S., Cyprys P., Gerlach M., Ochtrop P., Hackenberger C.P.R., Schumacher D. (2024). Design and evaluation of phosphonamidate-linked exatecan constructs for highly loaded, stable, and efficacious antibody-drug conjugates. Mol. Cancer Ther..

[19] Jin Y., Schladetsch M.A., Huang X., Balunas M.J., Wiemer A.J. (2022). Stepping forward in antibody-drug conjugate development. Pharmacol. Ther..

[20] Grairi M., Le Borgne M. (2024). Antibody-drug conjugates: Prospects for the next generation. Drug Discov. Ther..

[21] Ruan D.Y., Wu H.X., Meng Q., Xu R.H. (2024). Development of antibody-drug conjugates in cancer: Overview and prospects. Cancer Commun..

[22] Coussens L., Yang-Feng T.L., Liao Y.C., Chen E., Gray A., McGrath J., Seeburg P.H., Libermann T.A., Schlessinger J., Francke U. (1985). Tyrosine kinase receptor with extensive homology to EGF receptor shares chromosomal location with neu oncogene. Science.

[23] Hayes D.F. (2019). HER2 and breast cancer: A phenomenal success story. N. Engl. J. Med..

[24] Wells V., Mallucci L. (2009). Phosphoinositide 3-kinase targeting by the beta galactoside binding protein cytokine negates akt gene expression and leads aggressive breast cancer cells to apoptotic death. Breast Cancer Res..

[25] Kirouac D.C., Du J., Lahdenranta J., Onsum M.D., Nielsen U.B., Schoeberl B., McDonagh C.F. (2016). HER2+ cancer cell dependence on PI3K vs. MAPK signaling axes is determined by expression of EGFR, ERBB3 and CDKN1B. PLoS Comput. Biol..

[26] Koshkin V.S., Schafer J.M., Scherrer E., Boyiddle C., Schwartz N.R.M., Yu H., Fang Q., Baker A.F., Sayedian F.H., Chan E. (2025). Testing and Interpretation of Human Epidermal Growth Factor Receptor 2 Protein Expression and ERBB2 Gene Amplification in Advanced Urothelial Carcinoma. JCO Precis. Oncol..

[27] Yang M., Yao Y., Wang K., Qi L., Yang B., Khudadad M., Guo Y., Wang Y., Liu Y., Li L. (2026). Clinicopathological characteristics and prognostic significance of HER2 status evaluation in patients with urothelial carcinoma: A retrospective single-center experience in China. Virchows Arch..

[28] Jaehnig E.J., Fernandez-Martinez A., Vashist T.D., Holt M.V., Williams L., Lei J.T., Moon C.I., Kim B.J., Dou Y., Zhao H. (2025). Proteogenomic analysis of the CALGB 40601 (Alliance) HER2+ breast cancer neoadjuvant trial reveals resistance biomarkers. Cell Rep. Med..

[29] Munkácsy G., Santarpia L., Győrffy B. (2025). Exploring immune activation patterns in HER2-low and HER2-ultralow breast cancer subtypes. Oncologist.

[30] Bellmunt J., Werner L., Bamias A., Fay A.P., Park R.S., Riester M., Selvarajah S., Barletta J.A., Berman D.M., de Muga S. (2015). HER2 as a target in invasive urothelial carcinoma. Cancer Med..

[31] Slamon D.J., Clark G.M., Wong S.G., Levin W.J., Ullrich A., McGuire W.L. (1987). Human breast cancer: Correlation of relapse and survival with amplification of the HER-2/neu oncogene. Science.

[32] Ramtohul T., Djerroudi L., Lissavalid E., Nhy C., Redon L., Ikni L., Djelouah M., Journo G., Menet E., Cabel L. (2023). Multiparametric MRI and radiomics for the prediction of HER2-zero, -low, and -positive breast cancers. Radiology.

[33] Swain S.M., Shastry M., Hamilton E. (2023). Targeting HER2-positive breast cancer: Advances and future directions. Nat. Rev. Drug Discov..

[34] Jeong J., Shin J.H., Li W., Hong J.Y., Lim J., Hwang J.Y., Chung J.J., Yan Q., Liu Y., Choi J. (2021). MAL2 mediates the formation of stable HER2 signaling complexes within lipid raft-rich membrane protrusions in breast cancer cells. Cell Rep..

[35] Musolino A., Naldi N., Dieci M.V., Zanoni D., Rimanti A., Boggiani D., Sgargi P., Generali D.G., Piacentini F., Ambroggi M. (2008). Immunoglobulin G fragment C receptor polymorphisms and clinical efficacy of trastuzumab-based therapy in patients with HER-2/neu-positive metastatic breast cancer. J. Clin. Oncol..

[36] Barfield R.M., Kim Y.C., Chuprakov S., Zhang F., Bauzon M., Ogunkoya A.O., Yeo D., Hickle C., Pegram M.D., Rabuka D. (2020). A novel HER2-targeted antibody-drug conjugate offers the possibility of clinical dosing at trastuzumab-equivalent exposure levels. Mol. Cancer Ther..

[37] Pegram M.D., Miles D., Tsui C.K., Zong Y. (2020). HER2-overexpressing/amplified breast cancer as a testing ground for antibody-drug conjugate drug development in solid tumors. Clin. Cancer Res..

[38] Hamblett K.J., Jacob A.P., Gurgel J.L., Tometsko M.E., Rock B.M., Patel S.K., Milburn R.R., Siu S., Ragan S.P., Rock D.A. (2015). SLC46A3 is required to transport catabolites of noncleavable antibody maytansine conjugates from the lysosome to the cytoplasm. Cancer Res..

[39] Narayan P., Osgood C.L., Singh H., Chiu H.-J., Ricks T.K., Chow E.C.Y., Qiu J., Song P., Yu J., Namuswe F. (2021). FDA approval summary: Fam-trastuzumab deruxtecan-nxki for the treatment of unresectable or metastatic HER2-positive breast cancer. Clin. Cancer Res..

[40] Verma S., Miles D., Gianni L., Krop I.E., Welslau M., Baselga J., Pegram M., Oh D.-Y., Diéras V., Guardino E. (2012). Trastuzumab emtansine for HER2-positive advanced breast cancer. N. Engl. J. Med..

[41] Krop I.E., Kim S.-B., González-Martín A., LoRusso P.M., Ferrero J.-M., Smitt M., Yu R., Leung A.C.F., Wildiers H. (2014). Trastuzumab emtansine versus treatment of physician’s choice for pretreated HER2-positive advanced breast cancer (TH3RESA): A randomised, open-label, phase 3 trial. Lancet Oncol..

[42] Van Cutsem E., Bang Y.J., Feng-Yi F., Xu J.M., Lee K.W., Jiao S.C., Chong J.L., López-Sanchez R.I., Price T., Gladkov O. (2015). HER2 screening data from ToGA: Targeting HER2 in gastric and gastroesophageal junction cancer. Gastric Cancer.

[43] Cortés J., Diéras V., Lorenzen S., Montemurro F., Riera-Knorrenschild J., Thuss-Patience P., Allegrini G., De Laurentiis M., Lohrisch C., Oravcová E. (2020). Efficacy and safety of trastuzumab emtansine plus capecitabine vs trastuzumab emtansine alone in patients with previously treated ERBB2 (HER2)-positive metastatic breast cancer: A phase 1 and randomized phase 2 trial. JAMA Oncol..

[44] Peters S., Stahel R., Bubendorf L., Bonomi P., Villegas A., Kowalski D.M., Baik C.S., Isla D., Carpeno J.C., Garrido P. (2019). Trastuzumab emtansine (T-DM1) in patients with previously treated HER2-overexpressing metastatic non-small cell lung cancer: Efficacy, safety, and biomarkers. Clin. Cancer Res..

[45] Iwama E., Zenke Y., Sugawara S., Daga H., Morise M., Yanagitani N., Sakamoto T., Murakami H., Kishimoto J., Matsumoto S. (2022). Trastuzumab emtansine for patients with non-small cell lung cancer positive for human epidermal growth factor receptor 2 exon-20 insertion mutations. Eur. J. Cancer.

[46] Ferraro E., Drago J.Z., Modi S. (2021). Implementing antibody-drug conjugates (ADCs) in HER2-positive breast cancer: State of the art and future directions. Breast Cancer Res..

[47] Hurvitz S.A., Hegg R., Chung W.P., Im S.A., Jacot W., Ganju V., Chiu J.W.Y., Xu B., Hamilton E., Madhusudan S. (2023). Trastuzumab deruxtecan versus trastuzumab emtansine in patients with HER2-positive metastatic breast cancer: Updated results from DESTINY-Breast03, a randomised, open-label, phase 3 trial. Lancet.

[48] Conilh L., Fournet G., Fourmaux E., Murcia A., Matera E.L., Joseph B., Dumontet C., Viricel W. (2021). Exatecan Antibody Drug Conjugates Based on a Hydrophilic Polysarcosine Drug-Linker Platform. Pharmaceuticals.

[49] Nie C., Xu W., Guo Y., Gao X., Lv H., Chen B., Wang J., Liu Y., Zhao J., Wang S. (2023). Immune checkpoint inhibitors enhanced the antitumor efficacy of disitamab vedotin for patients with HER2-positive or HER2-low advanced or metastatic gastric cancer: A multicenter real-world study. BMC Cancer.

[50] Shitara K., Bang Y.J., Iwasa S., Sugimoto N., Ryu M.H., Sakai D., Chung H.C., Kawakami H., Yabusaki H., Sakamoto Y. (2024). Trastuzumab deruxtecan in HER2-positive advanced gastric cancer: Exploratory biomarker analysis of the randomized, phase 2 DESTINY-Gastric01 trial. Nat. Med..

[51] Van Cutsem E., di Bartolomeo M., Smyth E., Chau I., Park H., Siena S., Lonardi S., Wainberg Z.A., Ajani J., Chao J. (2023). Trastuzumab deruxtecan in patients in the USA and Europe with HER2-positive advanced gastric or gastroesophageal junction cancer with disease progression on or after a trastuzumab-containing regimen (DESTINY-Gastric02): Primary and updated analyses from a single-arm, phase 2 study. Lancet Oncol..

[52] Li B.T., Smit E.F., Goto Y., Nakagawa K., Udagawa H., Mazières J., Nagasaka M., Bazhenova L., Saltos A.N., Felip E. (2022). Trastuzumab deruxtecan in HER2-mutant non-small-cell lung cancer. N. Engl. J. Med..

[53] Goto K., Goto Y., Kubo T., Ninomiya K., Kim S.W., Planchard D., Ahn M.J., Smit E.F., de Langen A.J., Pérol M. (2023). Trastuzumab deruxtecan in patients with HER2-mutant metastatic non-small-cell lung cancer: Primary results from the randomized, phase II DESTINY-Lung02 trial. J. Clin. Oncol..

[54] Peng Z., Liu T., Wei J., Wang A., He Y., Yang L., Zhang X., Fan N., Luo S., Li Z. (2021). Efficacy and safety of a novel anti-HER2 therapeutic antibody RC48 in patients with HER2-overexpressing, locally advanced or metastatic gastric or gastroesophageal junction cancer: A single-arm phase II study. Cancer Commun..

[55] Sun L., Jia X., Wang K., Li M. (2024). Unveiling the future of breast cancer therapy: Cutting-edge antibody-drug conjugate strategies and clinical outcomes. Breast.

[56] Bashi A.C., Proia T.A., Lawson M., Nelson A., Ireland L., Randle S.J., Agrawal S., Rosen A., Carroll D., Mettetal J.T. (2026). Capivasertib combines with trastuzumab deruxtecan to enhance anti-tumour activity in HER2-positive and HER2-low tumours. Mol. Cancer Ther..

[57] Yaghoubi S., Gharibi T., Karimi M.H., Sadeqi Nezhad M., Seifalian A., Tavakkol R., Bagheri N., Dezhkam A., Abdollahpour-Alitappeh M. (2021). Development and biological assessment of MMAE-trastuzumab antibody-drug conjugates (ADCs). Breast Cancer.

[58] Shi F., Liu Y., Zhou X., Shen P., Xue R., Zhang M. (2022). Disitamab vedotin: A novel antibody-drug conjugate for cancer therapy. Drug Deliv..

[59] Jiang J., Dong L., Wang L., Wang L., Zhang J., Chen F., Zhang X., Huang M., Li S., Ma W. (2016). HER2-targeted antibody drug conjugates for ovarian cancer therapy. Eur. J. Pharm. Sci..

[60] Wang J., Liu Y., Zhang Q., Li W., Feng J., Wang X., Fang J., Han Y., Xu B. (2024). Disitamab vedotin, a HER2-directed antibody-drug conjugate, in patients with HER2-overexpression and HER2-low advanced breast cancer: A phase I/Ib study. Cancer Commun..

[61] Cullinane C., Fleming C., O’Leary D.P., Hassan F., Kelly L., O’Sullivan M.J., Corrigan M.A., Redmond H.P. (2020). Association of circulating tumor DNA with disease-free survival in breast cancer: A systematic review and meta-analysis. JAMA Netw. Open.

[62] Wang Y., Gong J., Wang A., Wei J., Peng Z., Wang X., Zhou J., Qi C., Liu D., Li J. (2024). Disitamab vedotin (RC48) plus toripalimab for HER2-expressing advanced gastric or gastroesophageal junction and other solid tumours: A multicentre, open label, dose escalation and expansion phase 1 trial. eClinicalMedicine.

[63] Qu F., Lu R., Wu X., Wu Q., Zhao M., Li H., Yuan Y., Han Z., Cai D., Huang X. (2024). Efficacy and safety of RC48-ADC in HER2-positive and HER2-low metastatic breast cancer: A multicenter, real-world study. Front. Oncol..

[64] Sheng X., Yan X., Wang L., Shi Y., Yao X., Luo H., Shi B., Liu J., He Z., Yu G. (2021). Open-label, multicenter, phase II study of RC48-ADC, a HER2-targeting antibody-drug conjugate, in patients with locally advanced or metastatic urothelial carcinoma. Clin. Cancer Res..

[65] Yao H., Yan M., Tong Z., Wu X., Ryu M.H., Kim J.H., Park J., Zhong Y., Han W., Liu C. (2023). Abstract CT175: Safety, tolerability, pharmacokinetics, and antitumor activity of SHR-A1811 in HER2-expressing/mutated advanced solid tumors: A global phase 1, multi-center, first-in-human study. Cancer Res..

[66] Turner N., Saura C., Aftimos P., van den Tweel E., Oesterholt M., Koper N., Colleoni M., Kaczmarek E., Punie K., Song X. (2025). Trastuzumab duocarmazine in pretreated human epidermal growth factor receptor 2–positive advanced or metastatic breast cancer: An open-label, randomized, phase III trial (TULIP). J. Clin. Oncol..

[67] Hartley J.A., Flynn M.J., Bingham J.P., Corbett S., Reinert H., Tiberghien A., Masterson L.A., Antonow D., Adams L., Chowdhury S. (2018). Pre-clinical pharmacology and mechanism of action of SG3199, the pyrrolobenzodiazepine (PBD) dimer warhead component of antibody-drug conjugate (ADC) payload tesirine. Sci. Rep..

[68] Corbett S., Huang S., Zammarchi F., Howard P.W., van Berkel P.H., Hartley J.A. (2020). The Role of Specific ATP-Binding Cassette Transporters in the Acquired Resistance to Pyrrolobenzodiazepine Dimer-Containing Antibody-Drug Conjugates. Mol. Cancer Ther..

[69] Phillips G.D.L., Li G., Dugger D.L., Crocker L.M., Parsons K.L., Mai E., Blättler W.A., Lambert J.M., Chari R.V., Lutz R.J. (2008). Targeting HER2-positive breast cancer with trastuzumab-DM1, an antibody-cytotoxic drug conjugate. Cancer Res..

[70] Yu J., Fang T., Yun C., Liu X., Cai X. (2022). Antibody-Drug Conjugates Targeting the Human Epidermal Growth Factor Receptor Family in Cancers. Front. Mol. Biosci..

[71] Sheng X., Wang L., He Z., Shi Y., Luo H., Han W., Yao X., Shi B., Liu J., Hu C. (2024). Efficacy and Safety of Disitamab Vedotin in Patients with Human Epidermal Growth Factor Receptor 2-Positive Locally Advanced or Metastatic Urothelial Carcinoma: A Combined Analysis of Two Phase II Clinical Trials. J. Clin. Oncol..

[72] Li J.J., Wang Z.H., Chen L., Zhang W.J., Ma L.X.X., Wu J., Liu G.Y., Hou Y.F., Yu K.D., Di G.H. (2025). Efficacy and safety of neoadjuvant SHR-A1811 with or without pyrotinib in women with locally advanced or early HER2-positive breast cancer: A randomized, open-label, phase II trial. Ann. Oncol..

[73] Xu Z., Guo D., Jiang Z., Tong R., Jiang P., Bai L., Chen L., Zhu Y., Guo C., Shi J. (2019). Novel HER2-Targeting Antibody-Drug Conjugates of Trastuzumab Beyond T-DM1 in Breast Cancer: Trastuzumab Deruxtecan(DS-8201a) and (Vic-)Trastuzumab Duocarmazine (SYD985). Eur. J. Med. Chem..

[74] Liu Y., Huang W., Saladin R.J., Hsu J.C., Cai W., Kang L. (2024). Trop-2-targeted molecular imaging in solid tumors: Current advances and future outlook. Mol. Pharm..

[75] El Sewedy T., Fornaro M., Alberti S. (1998). Cloning of the murine Trop-2 gene: Conservation of a PIP2-binding sequence in the cytoplasmic domain of Trop-2. Int. J. Cancer.

[76] Lipinski M., Parks D.R., Rouse R.V., Herzenberg L.A. (1981). Human trophoblast cell-surface antigens defined by monoclonal antibodies. Proc. Natl. Acad. Sci. USA.

[77] Zaman S., Jadid H., Denson A.C., Gray J.E. (2019). Targeting Trop-2 in solid tumors: Future prospects. OncoTargets Ther..

[78] Vidmar T., Pavšič M., Lenarčič B. (2013). Biochemical and preliminary X-ray characterization of the tumor-associated calcium signal transducer 2 (Trop-2) ectodomain. Protein Expr. Purif..

[79] Cubas R., Zhang S., Li M., Chen C., Yao Q. (2010). Trop-2 expression contributes to tumor pathogenesis by activating the ERK MAPK pathway. Mol. Cancer.

[80] Guerra E., Trerotola M., Tripaldi R., Aloisi A.L., Simeone P., Sacchetti A., Relli V., D’Amore A., La Sorda R., Lattanzio R. (2016). Trop-2 induces tumor growth through AKT and determines sensitivity to AKT inhibitors. Clin. Cancer Res..

[81] Hu Y., Zhu Y., Qi D., Tang C., Zhang W. (2024). Trop-2-targeted therapy in breast cancer. Biomark. Res..

[82] Stoyanova T., Goldstein A.S., Cai H., Drake J.M., Huang J., Witte O.N. (2012). Regulated proteolysis of Trop-2 drives epithelial hyperplasia and stem cell self-renewal via β-catenin signaling. Genes Dev..

[83] Tomiyama E., Fujita K., Hashimoto M., Adomi S., Kawashima A., Minami T., Yoshimura K., Uemura H., Nonomura N. (2022). Comparison of molecular profiles of upper tract urothelial carcinoma vs. urinary bladder cancer in the era of targeted therapy: A narrative review. Transl. Androl. Urol..

[84] Lombardi P., Filetti M., Falcone R., Altamura V., Paroni Sterbini F., Bria E., Fabi A., Giannarelli D., Scambia G., Daniele G. (2023). Overview of Trop-2 in cancer: From pre-clinical studies to future directions in clinical settings. Cancers.

[85] Dri A., Arpino G., Bianchini G., Curigliano G., Danesi R., De Laurentiis M., Del Mastro L., Fabi A., Generali D., Gennari A. (2024). Breaking barriers in triple-negative breast cancer (TNBC): Unleashing the power of antibody-drug conjugates (ADCs). Cancer Treat. Rev..

[86] Cheng W., Liu D., Guo M., Li H., Wang Q. (2022). Sophoraflavanone G suppresses the progression of triple-negative breast cancer via the inactivation of EGFR-PI3K-AKT signaling. Drug Dev. Res..

[87] Thomas J., Sun M., Getz T., Ho B., Nauseef J.T., Tagawa S.T. (2023). Antibody-drug conjugates for urothelial carcinoma. Urol. Oncol..

[88] Rugo H.S., Tolaney S.M., Loirat D., Punie K., Bardia A., Hurvitz S.A., O’Shaughnessy J., Cortés J., Diéras V., Carey L.A. (2022). Safety analyses from the phase 3 ASCENT trial of sacituzumab govitecan in metastatic triple-negative breast cancer. npj Breast Cancer.

[89] Qiu S., Zhang J., Wang Z., Lan H., Hou J., Zhang N., Wang X., Lu H. (2023). Targeting Trop-2 in cancer: Recent research progress and clinical application. BBA Rev. Cancer.

[90] Okajima D., Yasuda S., Maejima T., Karibe T., Sakurai K., Aida T., Toki T., Yamaguchi J., Kitamura M., Kamei R. (2021). Datopotamab deruxtecan, a novel Trop-2-directed antibody-drug conjugate, demonstrates potent antitumor activity by efficient drug delivery to tumor cells. Mol. Cancer Ther..

[91] Royce M., Shah M., Zhang L., Cheng J., Bonner M.K., Pegues M., Miller C.P., Leu L., Price L.S.L., Qiu J. (2025). FDA Approval Summary: Datopotamab Deruxtecan-dlnk for Treatment of Patients with Unresectable or Metastatic, HR-Positive, HER2-Negative Breast Cancer. Clin. Cancer Res..

[92] Zhou D.D., Zhai X.T., Zhang L.W., Xie Z.H., Wang Y., Zhen Y.S., Gao R.J., Miao Q.F. (2024). A new TROP2-targeting antibody-drug conjugate shows potent antitumor efficacy in breast and lung cancers. npj Precis. Oncol..

[93] Strop P., Tran T.T., Dorywalska M., Delaria K., Dushin R., Wong O.K., Ho W.H., Zhou D., Wu A., Kraynov E. (2016). RN927C, a Site-Specific Trop-2 Antibody-Drug Conjugate (ADC) with Enhanced Stability, Is Highly Efficacious in Preclinical Solid Tumor Models. Mol. Cancer Ther..

[94] Ma D., Dai L.-J., Wu X.-R., Liu C.-L., Zhao S., Zhang H., Chen L., Xiao Y., Li M., Zhao Y.-Z. (2025). Spatial determinants of antibody-drug conjugate SHR-A1811 efficacy in neoadjuvant treatment for HER2-positive breast cancer. Cancer Cell.

[95] Notini G., Galbardi B., Viale G., Naldini M.M., Bosi C., De Micheli G., Licata L., Mariani M., Piras M., Criscitiello C. (2026). Pan-cancer multi-omic integration of Trop2 reveals biological determinants and translational implications for ADC therapy. npj Precis. Oncol..

[96] Morgenstern-Kaplan D., Kareff S.A., Trabolsi A., Rodriguez E., Krause H., Ribeiro J.R., Tan H., Antonarakis E.S., Lou E., Nagasaka M. (2024). Genomic, immunologic, and prognostic associations of TROP2 (TACSTD2) expression in solid tumors. Oncologist.

[97] Xu S., Wang Y.L. (2019). Lidamycin-based targeted drugs for tumor therapy. Chin. Bull. Life Sci..

[98] Rampa D.R., Seo M., Ogata N., Yang Z., Sridhar N., Fujii T., Wannaphut C., Maynard J.A., Tsuchikama K., Sledge G.W. (2026). Payload Diversification Overcomes Resistance and Guides Sequential Antibody-Drug Conjugate Therapy in Breast Cancer. Clin. Cancer Res..

[99] Heist R.S., Sands J., Bardia A., Shimizu T., Lisberg A., Krop I., Yamamoto N., Kogawa T., Al-Hashimi S., Fung S.S.M. (2024). Clinical management, monitoring, and prophylaxis of adverse events of special interest associated with datopotamab deruxtecan. Cancer Treat. Rev..

[100] Tong Y., Fan X., Liu H., Liang T. (2024). Advances in Trop-2 targeted antibody-drug conjugates for breast cancer: Mechanisms, clinical applications, and future directions. Front. Immunol..

[101] Buck S.A.J., Koolen S.L.W., Mathijssen R.H.J., de Wit R., van Soest R.J. (2021). Cross-resistance and drug sequence in prostate cancer. Drug Resist. Updates.

[102] Mai N., Lieberman M.M.K., Ferraro E., Bromberg M., Chen Y., Razavi P., Modi S., Chandarlapaty S., Walsh E.M., Drago J.Z. (2025). Sequential Antibody-Drug Conjugate Therapy in Patients with Metastatic Breast Cancer Treated with Sacituzumab Govitecan and Trastuzumab Deruxtecan. JCO Precis. Oncol..

[103] Liu Y., Liu Y., Wu T., Bai X., Wang S., Zhang L., Fu Z., Shi C. (2026). Drug resistance to antibody-drug conjugates: Mechanisms, challenges, and perspectives. Cancer Biol. Med..

[104] Yazdi N., Pourjamal N., Katainen R., Väänänen J., Dai J., Vähärautio A., Isola J., Kempas M., Le Joncour V., Laakkonen P. (2025). Drug-tolerant persisting polyploid giant cancer cells mediate resistance to HER2-targeting antibody-drug conjugates. Cancer Lett..

[105] Cerci B., Saatci O., Basik M., Sahin O. (2026). Mechanisms of resistance to antibody-drug conjugates in breast cancer. Drug Resist. Updates.

[106] Zhou K., Liu X., Zhu H. (2025). Overcoming resistance to antibody-drug conjugates: From mechanistic insights to cutting-edge strategies. J. Hematol. Oncol..

[107] Alradwan I.A., Alnefaie M.K., Al Fayez N., Aodah A.H., Majrashi M.A., Alturki M., Fallatah M.M., Almughem F.A., Tawfik E.A., Alshehri A.A. (2025). Strategic and Chemical Advances in Antibody-Drug Conjugates. Pharmaceutics.

[108] D’Agostino E., Pirola M., Callegari V., Tonni E., Tchawa C., Matranga R., Ponzoni O., Oltrecolli M., Piombino C., Pipitone S. (2025). Future directions for antibody-drug conjugates in urothelial cancer. Expert Rev. Anticancer Ther..

[109] Sabit H., Abbas S., El-Safoury M.T., Madkour E.M., Mahmoud S., Abdel-Ghany S., Albrahim Y., Al-Dhuayan I.S., Rashwan S., El-Hashash A. (2025). Antibody-Drug Conjugates in Breast Cancer: Navigating Innovations, Overcoming Resistance, and Shaping Future Therapies. Biomedicines.

[110] Xu K., Bayani J., Mallon E., Pond G.R., Piper T., Hasenburg A., Markopoulos C.J., Dirix L., Seynaeve C.M., van de Velde C.J.H. (2022). Discordance between Immunohistochemistry and Erb-B2 Receptor Tyrosine Kinase 2 mRNA to Determine Human Epidermal Growth Factor Receptor 2 Low Status for Breast Cancer. J. Mol. Diagn..

